# A central role for PBP2 in the activation of peptidoglycan polymerization by the bacterial cell elongation machinery

**DOI:** 10.1371/journal.pgen.1007726

**Published:** 2018-10-18

**Authors:** Patricia D. A. Rohs, Jackson Buss, Sue I. Sim, Georgia R. Squyres, Veerasak Srisuknimit, Mandy Smith, Hongbaek Cho, Megan Sjodt, Andrew C. Kruse, Ethan C. Garner, Suzanne Walker, Daniel E. Kahne, Thomas G. Bernhardt

**Affiliations:** 1 Department of Microbiology and Immunobiology, Harvard Medical School, Boston, Massachusetts, United States of America; 2 Department of Molecular and Cellular Biology, Harvard University, Cambridge, Massachusetts, United States of America; 3 Department of Chemistry and Chemical Biology, Harvard University, Cambridge, Massachusetts, United States of America; 4 Department of Biological Sciences, Sungkyunkwan University, Suwon, Gyeonggi, Korea; 5 Department of Biological Chemistry and Molecular Pharmacology, Harvard Medical School, Boston, Massachusetts, United States of America; 6 Howard Hughes Medical Institute, Boston, Massachusetts, United States of America; University of Geneva Medical School, SWITZERLAND

## Abstract

Cell elongation in rod-shaped bacteria is mediated by the Rod system, a conserved morphogenic complex that spatially controls cell wall assembly by the glycan polymerase RodA and crosslinking enzyme PBP2. Using *Escherichia coli* as a model system, we identified a PBP2 variant that promotes Rod system function when essential accessory components of the machinery are inactivated. This PBP2 variant hyperactivates cell wall synthesis in vivo and stimulates the activity of RodA-PBP2 complexes in vitro. Cells with the activated synthase also exhibited enhanced polymerization of the actin-like MreB component of the Rod system. Our results define an activation pathway governing Rod system function in which PBP2 conformation plays a central role in stimulating both glycan polymerization by its partner RodA and the formation of cytoskeletal filaments of MreB to orient cell wall assembly. In light of these results, previously isolated mutations that activate cytokinesis suggest that an analogous pathway may also control cell wall synthesis by the division machinery.

## Author summary

The cell wall of bacteria determines their shape and protects them from osmotic lysis. Two enzymatic activities are required for cell wall synthesis: glycan polymerization and crosslinking. A major new family of glycan polymerases was recently discovered and was proposed to work in complex with crosslinking enzymes called penicillin-binding proteins (PBPs). How the activities of these enzymes are coordinated to prevent the toxic generation of uncrosslinked glycans has remained unknown. Our analysis of the cell elongation system of *Escherichia coli* has revealed that this coupling is mediated by changes in the PBP that activate glycan chain synthesis by the polymerase. Furthermore, we present genetic evidence that this activation event is mediated by a component of the elongation machinery with a previously unknown function. Discovery of this activation pathway provides new mechanistic insight into the cell wall biogenesis process and identifies a new avenue to disrupt it for antibiotic development.

## Introduction

Bacterial cells typically surround themselves with a cell wall exoskeleton made of the heteropolymer peptidoglycan (PG). This structure is essential for cell integrity and understanding its biogenesis is of great practical significance because the pathway is a proven target for many of our most effective antibiotic therapies [1]. The PG layer is also the major determinant of bacterial cell shape such that studies of PG assembly are also of fundamental importance for determining the mechanisms responsible for bacterial growth and morphogenesis [2].

PG is composed of long glycan strands with a disaccharide repeating unit of N-acetylmuramic acid (MurNAc)-β-1-4-N-acetylglucosamine (GlcNAc) and a pentapeptide stem attached to the MurNAc sugar [3]. The strands are polymerized by membrane-embedded PG glycosyltransferase (PGTase) enzymes using the lipid-linked disaccharide-pentapeptide precursor called lipid II. The polymerized glycans are then crosslinked via the formation of amide bonds between attached peptides by transpeptidase (TPase) enzymes. Several different types of synthases with these activities work together to build what ultimately becomes a cell-shaped polymer matrix that envelops the cytoplasmic membrane and protects it from osmotic lysis.

To direct PG matrix assembly during cell growth and division, most rod-shaped bacteria employ two multi-protein synthetic machineries organized by cytoskeletal filaments [2]. The Rod system (elongasome) utilizes the actin-like MreB protein to promote cell elongation and maintain cell shape, whereas the cytokinetic ring (divisome) uses the tubulin-like FtsZ protein to orchestrate cell division and the construction of the daughter cell poles. For many years, the main PG synthases of these machineries were thought to be the class A penicillin-binding proteins (aPBPs) [2]. These bifunctional synthases possess both PGT and TP activity to make PG, and until recently, the PGT domain of aPBPs was the only known family of PG polymerases. This view of PG biogenesis was called into question by the discovery of PG polymerase activity for the SEDS (shape, elongation, division, and sporulation) family protein RodA of the Rod system [4].

SEDS family proteins are widely distributed in bacteria [4,5] and are known to form complexes with class B PBPs (bPBPs) [6,7], which are monofunctional TPases only thought to be capable of PG crosslinking. Thus, SEDS-bPBP complexes have been proposed to represent a second type of PGT/TP enzymatic system for PG synthesis, with FtsW-PBP3 and RodA-PBP2 functioning as the SEDS-bPBP pairs for the divisome and Rod system, respectively [4,8]. Although it remains possible that the SEDS-bPBP synthases work together with aPBPs in the same complexes, functional and localization studies suggest otherwise [8]. In both *Escherichia coli* and *Bacillus subtilis*, the aPBPs have been shown to display distinct subcellular localization dynamics from Rod system components and to be dispensable for the activity of the machinery [4,8]. It has therefore been proposed that a RodA-PBP2 complex forms the core PG synthase of the Rod system, an idea supported by recent evolutionary co-variation analysis [9], and the finding that the aPBPs largely operate outside of the cytoskeletal system during cell elongation [8]. A similar division of labor between aPBPs and FtsW-PBP3 may also be taking place during cytokinesis, but the relative contributions of the two types of synthases to the division process requires further definition.

The discovery that RodA is a PG polymerase raises many important questions about the function of the Rod system. Is the polymerase activity of this new synthase regulated, and if so, how is its activity controlled to maintain a uniform rod shape? Does RodA work with PBP2 as proposed, and if so, how is the polymerase activity of RodA coordinated with the crosslinking activity of PBP2? Coupling of these activities is expected to be critical because its disruption by beta-lactam antibiotics is part of the lethal mechanism of action of these drugs [10]. For example, the beta-lactam mecillinam blocks the TP activity of PBP2 while leaving the activity of RodA unaffected. As a result, RodA generates uncrosslinked glycan strands that are rapidly degraded, causing a futile cycle of PG synthesis and degradation that is cytotoxic [8,10]. Thus, during its normal function, the Rod system is likely to possess a fail-safe that prevents RodA from initiating PG polymerization unless it is engaged with PBP2 to crosslink its product glycans. Finally, aside from MreB, RodA, and PBP2, the Rod system typically includes the additional proteins MreC, MreD, and RodZ. Despite their broad conservation throughout cell wall producing bacteria, even in non-rod-shaped organisms lacking MreB [11], the function of these additional Rod system components remains unclear.

In this report, we describe the discovery of PBP2 variants that suppress the growth and shape defects of *mreC* hypomorphs. One of the altered PBP2 variants was shown to hyperactivate cell wall synthesis by the Rod system in vivo and to stimulate the polymerase activity of RodA-PBP2 complexes in vitro. Furthermore, studies of Rod system localization dynamics in the mutant cells indicate that the PBP2 variant promotes the formation of active Rod complexes by enhancing MreB filament formation. Overall, our results define an activation pathway for the cell elongation machinery in which PBP2 plays a central role in both stimulating PG polymerization by RodA and modulating MreB filament formation to orient new synthesis [12]. This mode of activation provides a built-in mechanism for coupling cell wall polymerization and crosslinking to prevent the toxic accumulation of uncrosslinked glycans. Moreover, the phenotypes of previously described cell division mutants [13–15] and our recent biochemical studies of FtsW in complex with its cognate bPBP [16] suggest that this activation pathway is conserved to control PG synthesis by the divisome.

## Results

### A strategy to identify mutants with hyperactive Rod systems

In *E*. *coli* and other organisms, each protein within the Rod system is required for proper functioning of the complex [11,17–22]. Rod system defects result in a loss of rod shape and cell death under typical growth conditions, but spherical *E*. *coli* Rod^-^ mutants can survive on minimal medium at low temperatures [18]. Thus, mutants inactivated for the Rod system can be constructed under permissive conditions (minimal medium) and suppressors of these defects can be isolated by plating the mutants on rich medium (non-permissive conditions) and selecting for growth. Starting with a Δ*rodZ* mutant background, this suppressor isolation strategy has been successfully used to investigate how the interaction between RodZ and MreB may modulate Rod system function [23,24]. We reasoned that similar selections for suppressors of other Rod system defects might help us understand how the PG synthetic enzymes within the complex are controlled.

**Fig 1:**
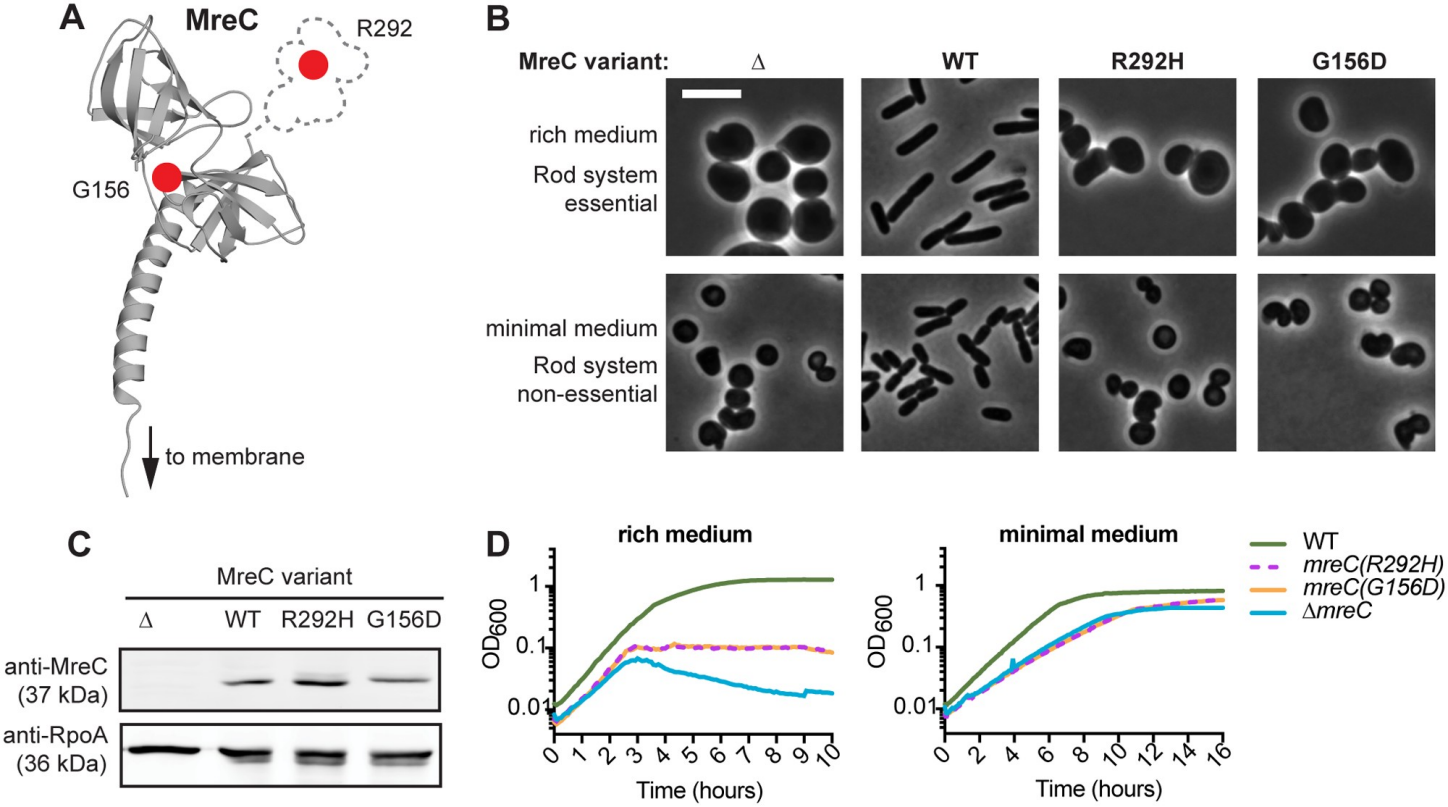
Amino acid substitutions in MreC affect protein function but not stability. **A.** Structure of *E*. *coli* MreC, modeled on the *L*. *monocytogenes* MreC structure using Phyre2 [25,26]. The dotted area indicates the C-terminal domain of unknown structure found in the E. coli protein (AA 273–367) but absent from the *L*. *monocytogenes* protein. Locations of the amino acid substitutions affecting function are indicated by the red dots. **B.** Strains containing the indicated *mreC* missense alleles at the native genomic locus [MT4, HC555, PR5, PR30] were grown overnight in M9 medium supplemented with 0.2% casamino acids and 0.2% glucose (M9 CAA glu), diluted to OD_600_ = 0.05 in the same medium, and grown at 30°C until the OD_600_ reached 0.2. The resulting cultures were then either diluted to OD_600_ = 0.025 in M9 CAA glu, or gently pelleted and resuspended in LB to an OD_600_ = 0.025. Cells were grown at 30°C to an OD_600_ of 0.2. At this time, cells were fixed, immobilized, and imaged using phase-contrast microscopy. Scale bar, 5 μm. **C.** Immunoblot detecting MreC and the loading control RpoA. Each lane was loaded with 5 μg of total protein from whole-cell extracts prepared from cultures of the above strains grown in M9 CAA glu (OD_600_ ~ 0.3). **D.** Growth curves of the above strains grown in either M9 CAA glu or LB at 30°C. Prior to beginning the growth measurements, cells were grown to OD_600_ = 0.2 in M9 CAA glu at 30°C, then either diluted to OD_600_ = 0.025 in M9 CAA glu, or gently pelleted and resuspended in LB to an OD_600_ = 0.025.

Defects resulting from a missense mutation are expected to be easier for cells to overcome in a suppressor selection than those due to a deletion allele. We therefore developed a strategy to rapidly identify missense alleles in Rod system genes that result in a stable yet defective gene product. In a report that will be published separately, we applied this method to *mreC*. Several defective *mreC* alleles were identified. The two mutants displaying the most severe defects encoded MreC proteins with a G156D or an R292H substitution (Fig 1A). When the *mreC(G156D)* or *mreC(R292H)* alleles were constructed at the native *mre* locus, the resulting cells had a spherical morphology and failed to grow in rich medium (LB), reminiscent of an *mreC* deletion (Fig 1B and 1D). Although stable MreC protein accumulated in these mutants (Fig 1C), the proteins were incapable of promoting Rod system activity. We therefore concluded that the MreC variants identified were functionally defective and therefore suitable for use in a suppressor analysis.

Cells harboring the *mreC(G156D)* or *mreC(R292H)* alleles were plated on rich medium, the non-permissive condition for mutants defective for Rod system activity. Suppressors restoring growth arose at a frequency of 10^−5^ (S1 Table). Many of these isolates remained spherical, indicating that they had likely acquired mutations that allow spheres to grow on rich medium. However, additional screening identified several isolates that grew with a long axis, indicating at least a partial restoration of rod shape. Of these suppressors, two displayed near normal rod shape and were chosen for further analysis.

### Amino acid substitutions in PBP2 suppress MreC defects

**Fig 2:**
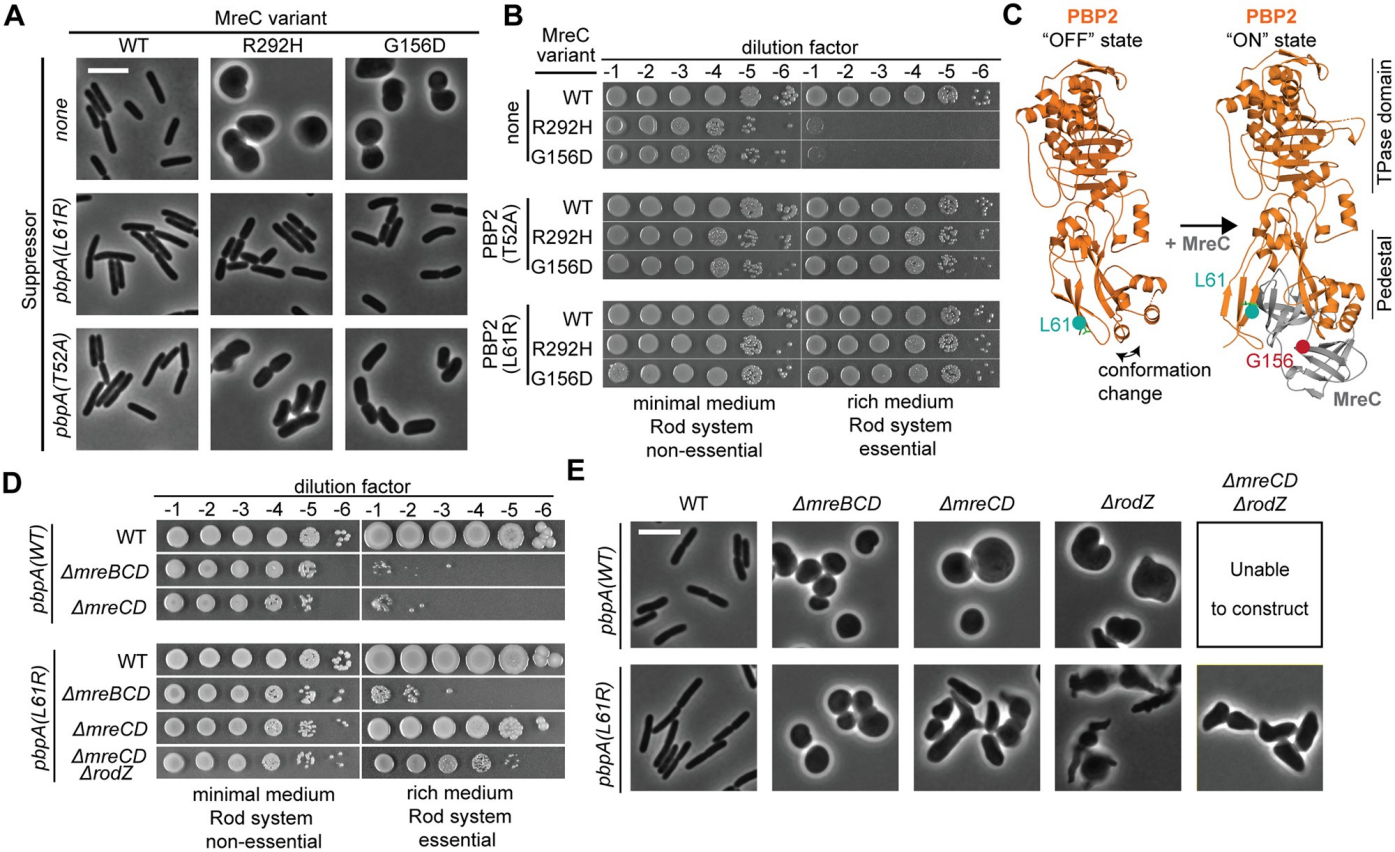
Substitutions in PBP2 suppress the growth and shape phenotypes of Rod system mutants. **A.** Strains containing the indicated point mutations at the native genomic locus [PR164, PR165, PR166, PR127, PR128, PR129, PR131, PR124, PR125] were grown overnight in M9 CAA glu, diluted to OD_600_ = 0.05 in the same medium, and grown to exponential phase (OD_600_ = 0.2). Cells were gently pelleted, resuspended, and diluted in LB (OD_600_ = 0.025), then grown until the OD_600_ reached 0.2. At this time, cells were fixed, immobilized, and imaged using phase-contrast microscopy. All growth was at 30°C. Scale bar, 5 μm. **B.** Overnight cultures of the above strains were serially diluted and spotted on either M9 CAA glu agar (Rod non-essential) or LB agar (Rod essential). Plates were incubated at 30°C for either 40 h (M9) or 16 h (LB) prior to being photographed. **C.** Shown are *E*. *coli* PBP2 and PBP2-MreC structures modeled from PDB-5LP4 and PDB-5LP5 [27] using Phyre 2 [26]. PBP2 is orange with residue L61 in turquoise. MreC is gray with residue G156 in red. Structural information is lacking for the juxta-membrane region of PBP2 containing residue T52, and for the C-terminal domain of MreC containing residue R292 **D.** Strains containing the indicated mutations were grown and spotted as in (B) [Top to bottom: PR132, PR136, PR137, PR78, PR129, PR140, PR149]. **E.** The above strains were grown and prepared for phase-contrast microscopy as described in (A). Scale bar, 5 μm.

Whole-genome sequencing was used to map the location of the *mreC* suppressors. Both isolates harbored mutations in the *pbpA* (*mrdA*) gene encoding PBP2, the PG crosslinking enzyme of the Rod system. Although the *pbpA(T52A)* allele was originally found to suppress *mreC(G156D)* and the *pbpA(L61R)* allele was first isolated as a suppressor of *mreC(R292H)*, neither suppressor was allele specific. Both were capable of suppressing the shape and viability defects of either *mreC* allele when the mutants were reconstructed in an otherwise normal parental strain background (Fig 2A and 2B). However, *pbpA(L61R)* was more robust at restoring normal rod shape than the *pbpA(T52A)* allele.

The changes in the altered PBP2 derivatives map to the membrane proximal region of the protein often referred to as the pedestal or non-penicillin-binding domain (Fig 2C). In the solved structures of bPBPs [28–30], this region consists of two interacting subdomains connected by a third subdomain forming a hinge that sits just underneath the catalytic TP domain. In a recently solved structure of an MreC-PBP2 complex from *Helicobacter pylori*, MreC interacts with the pedestal domain of PBP2 and in doing so causes its two interacting subdomains to swing open [27] (Fig 2C). The alterations in PBP2 that suppress the MreC defects are not predicted to be at locations directly involved in the PBP2-MreC interface. Moreover, PBP2 derivatives with changes in the same region, PBP2(Q51L) and PBP2(T52N), were previously shown to suppress a Rod system defect caused by a Δ*rodZ* mutation [24]. We therefore hypothesized that the conformational change in PBP2 induced by MreC may be part of a mechanism controlling PG synthesis by the core enzymatic components of the Rod system. We further reasoned that the PBP2 variants we identified might spontaneously achieve an activated conformation that stimulates PG polymerization and crosslinking such that they bypass the normal requirement for MreC and other components of the Rod machinery that may have regulatory functions.

To begin testing our hypothesis, we assessed whether the strongest suppressor of *mreC* missense mutations, PBP2(L61R), could also suppress the shape and viability defects of mutants deleted for Rod system genes. This variant suppressed the growth defect of Δ*rodZ* cells and partially restored their shape as expected based on its similarity to previously isolated Δ*rodZ* suppressors [24] (Fig 2E). PBP2(L61R) also had the additional ability to suppress the growth defect of a Δ*mreCD* mutant and a Δ*mreCD* Δ*rodZ* triple mutant (Fig 2D and 2E). Although rod shape was not fully restored in these cells, they displayed a long axis indicative of at least partial restoration of Rod system function (Fig 2E). Notably, this PBP2 variant was incapable of suppressing the shape or viability defects of a Δ*mreBCD* mutation, even when the *mreCD* genes were expressed in trans (Fig 2D, S1 Fig), indicating that the actin-like MreB protein remains essential for Rod system function in cells producing this altered PBP. These results are consistent with PBP2(L61R) adopting an activated conformation that mimics that induced upon assembly of the complete Rod system. Furthermore, the observation that partial rod shape can be restored with just MreB, RodA, and a PBP2 variant suggests that these three proteins form the minimal and essential core of the system in cells that require MreB for rod shape.

### PBP2(L61R) activates cell wall synthesis by the Rod system

**Fig 3:**
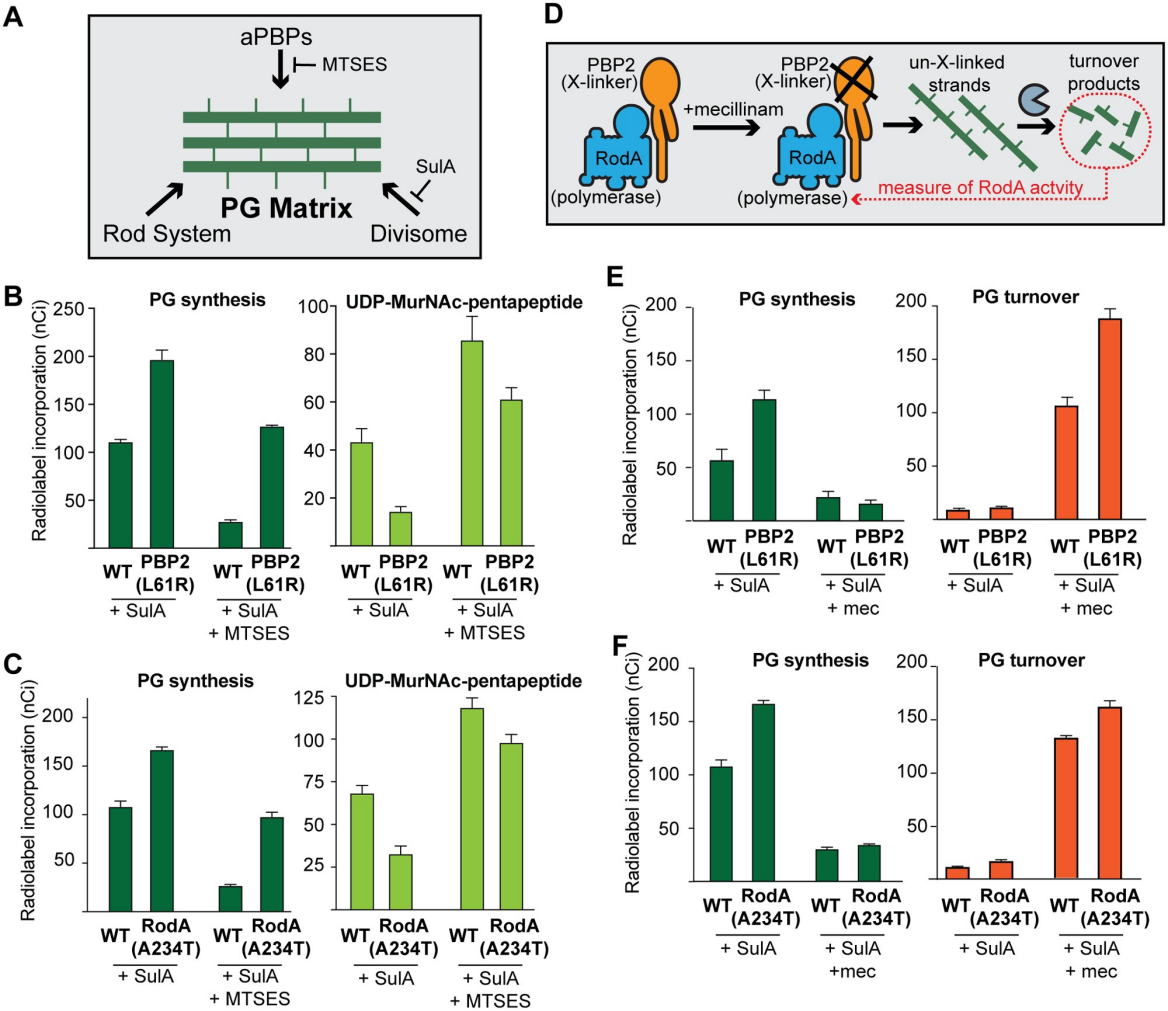
Cells expressing PBP2(L61R) or RodA(A234T) synthesize more peptidoglycan than wild type. **A.** Schematic of treatments used to inhibit specific PG synthesis systems during labeling experiments. In the labeling strains all aPBPs have been deleted (Δ*mtgA* Δ*mrcA* Δ*pbpC*) except for PBP1b, in which a cysteine mutation residue has been engineered near the active site (*mrcB(S247C)*), rendering it sensitive to MTSES. Labeling strains also contain mutations to block peptidoglycan recycling (Δ*ampD*) or mDAP conversion to lysine (Δ*lysA*), and a chromosomal-integrated expression construct to produce the FtsZ inhibitor SulA under inducible control (*attHKHC859*). **B.** Labeling strains producing PBP2(WT) or PBP2(L61R) from the native genomic locus [PR116(attHKHC859) and PR117(attHKHC859)] were pre-treated with 1.5 mM IPTG to induce SulA production and 1 mM MTSES, as indicated. Strains were then pulse-labeled with [^3^H]-mDAP, and peptidoglycan precursors (UDP-MurNAc-pentapeptide) and synthesis were measured. Results are the average of three independent experiments. Error bars represent the standard error of the mean. **C.** The same experiments and analysis as in (B) were performed using labeling strains producing RodA(WT) or RodA(A234T) from the native genomic locus [PR146(attHKHC859) and PR147(attHKHC859)]. **D.** Schematic illustrating the mechanism of turnover-product production upon beta-lactam treatment. **E.** Labeling strains producing PBP2(WT) or PBP2(L61R) from the native genomic locus [PR116(attHKHC859) and PR117(attHKHC859)] were pre-treated with 1.5 mM IPTG to induce SulA production. The indicated samples were also pre-treated with 10 μg/mL mecillinam. Strains were then pulse-labeled with [^3^H]-mDAP, and peptidoglycan synthesis and turnover products (anhydroMurNAC-tripeptide and -pentapeptide) were measured. Results are the average of four independent experiments. Note that a different stock of [^3^H]-mDAP was used for these experiments than in other panels such that total labeling observed was lower. **F.** The same experiments and analysis as in (E) were performed using labeling strains encoding RodA(WT) or RodA(A234T) at the native genomic locus [PR146(attHKHC859) and PR147(attHKHC859)]. Results are the average of three independent experiments.

To determine whether the L61R variant of PBP2 generally promotes Rod system activity, the *pbpA(L61R)* allele was engineered into *E*. *coli* cells with an otherwise normal complement of Rod system components. The growth rate of these cells was indistinguishable from that of wild type in both rich and minimal medium (S2 Table). However, the PBP2(L61R) cells were ~20% longer and ~10% thinner than cells with PBP2(WT) (S2 Table), providing an early indication that the Rod system may be activated by the altered PBP2 [31]. To monitor Rod system activity more directly, we followed cell wall synthesis in cells radiolabeled with [^3^H]-*meso*-diaminopimelic acid (mDAP), an amino acid unique to the PG stem peptide. For these studies, we used a previously described genetic background in which the divisome can be inactivated by an inducible copy of the FtsZ antagonist SulA and aPBP activity can be inhibited by the thiol-reactive reagent (2-sulfanatoethyl)methanethiosulfonate (MTSES) [8]. Thus, when SulA is produced and MTSES is added, radiolabel incorporation is mediated principally by the Rod system and thus reflects its activity (Fig 3A).

Following divisome inhibition, PBP2(L61R) cells synthesized PG at approximately twice the rate of wild-type cells (197 ± 10 nCi vs. 111 ± 2 nCi over ten minutes, p < 0.0001, Fig 3B). This increased synthesis activity was retained upon MTSES inhibition of the aPBPs, indicating that it indeed reflected elevated PG incorporation by the Rod system (127 ± 1 nCi vs. 37.1 ± 0.3 nCi over 10 minutes, p < 0.0001, Fig 3B). The increase radiolabel incorporation into PG was also accompanied by a corresponding decrease in the labeled pool of the precursor UDP-MurNAc-pentapeptide, indicating that flux through the PG synthesis pathway is likely increased in the PBP2(L61R) cells (Fig 3B, **right**). Immunoblot analysis and labeling with the fluorescent penicillin derivative Bocillin failed to detect any changes in MreB or PBP2 levels in cells harboring the altered PBP2 protein (S2 Fig). We therefore conclude that PBP2(L61R) is most likely activating PG synthesis by stimulating the activity of the Rod system.

### Rod system activation involves the stimulation of PG polymerization by RodA

**Fig 4:**
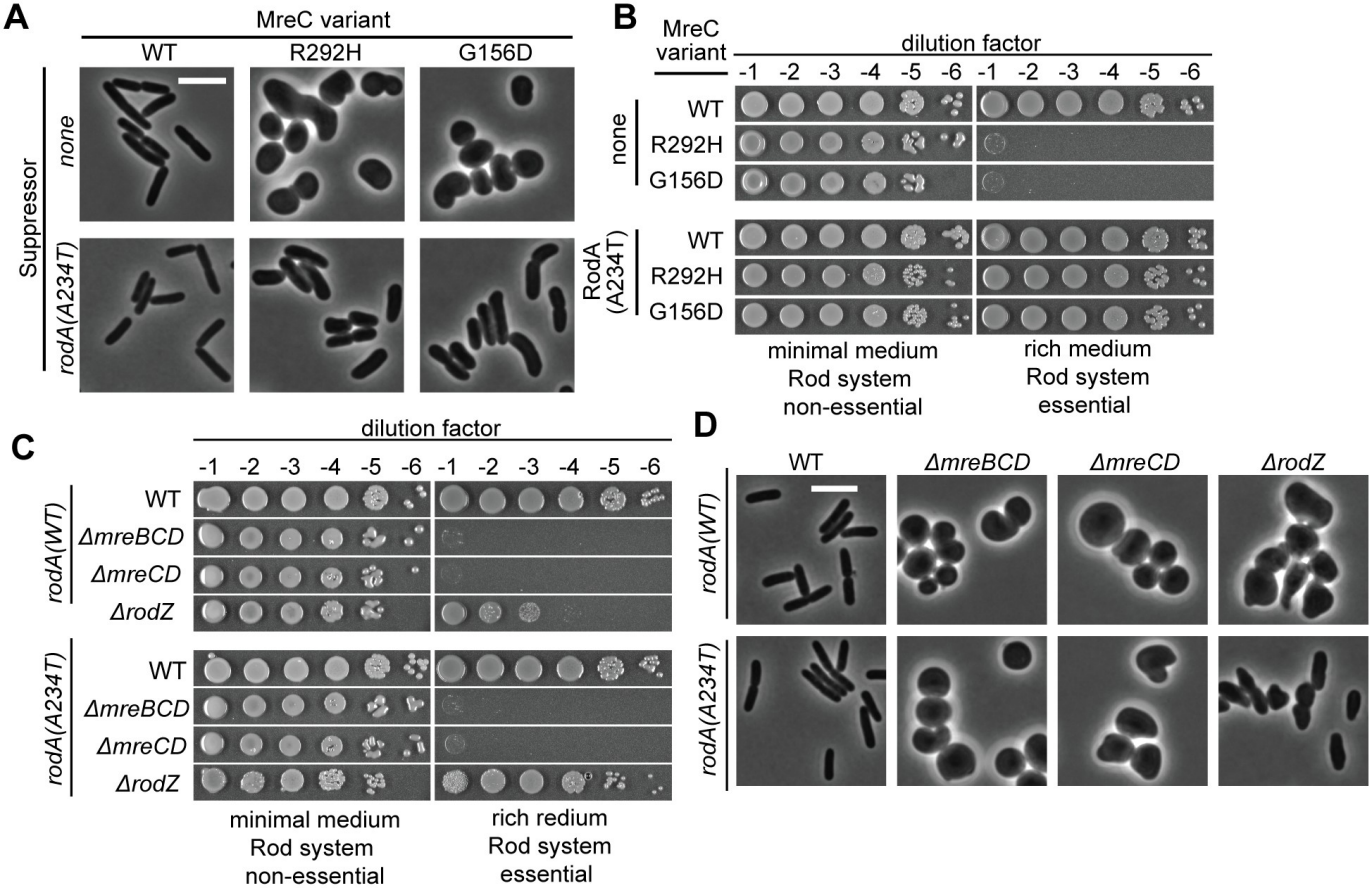
RodA(A234T) suppresses *mreC* missense mutants and Δ*rodZ* but not Δ*mreCD*. **A.** Strains containing the indicated missense mutations at the native genomic locus [PR158, PR159, PR160, PR161, PR162, PR163] were grown and imaged as in Fig 2A. **B.** Overnight cultures of the above strains were serially diluted and spotted on either M9 CAA glu agar (Rod non-essential) or LB agar (Rod essential). Plates were incubated at 30°C for either 40 h (M9) or 16 h (LB) before imaging. **C.** Overnight cultures of the indicated strains [PR150, PR152, PR153, PR154, PR151, PR155, PR156, PR157] were serially diluted and spotted as in Fig 3B. **D.** The indicated strains were grown, fixed, and imaged as described in Fig 2A.

In addition to changes in PBP2, RodA variants RodA(A234T) and RodA(T249P) were also previously identified as suppressors of a Δ*rodZ* mutation [24]. The *rodA(A234T)* mutant was reconstructed at its native locus and this suppression activity was confirmed. The change in RodA was also found to be capable of suppressing the growth and shape defects of *mreC(G156D)* and *mreC(R292H)* mutants (Fig 4A and 4B). However, RodA(A234T) could not compensate for the deletion of Rod system genes other than *rodZ*, indicating that it is not as potent of a suppressor as PBP2(L61R) (Fig 4C and 4D). Nevertheless, the suppression results suggested that RodA(A234T) is also capable of activating PG synthesis by the Rod system. We therefore monitored PG synthesis in *rodA(A234T)* mutant cells and found that Rod system activity was indeed enhanced relative to wild-type (167 ± 3 nCi vs. 108 ± 6 over ten minutes, p = 0.001, Fig 3C). In line with the relative suppression power of the variants, the observed PG synthesis activation by RodA(A234T) was not as great as that observed in cells producing PBP2(L61R).

Based on the ability of RodA and PBP2 variants to stimulate PG synthesis by the Rod system we hypothesized that activation in both cases may ultimately result from the enhancement of PG polymerization by RodA. To test this possibility more directly, we used a modified radiolabeling assay in which the beta-lactam mecillinam was included. Mecillinam specifically blocks the TP activity of PBP2 but allows continued glycan polymerization by RodA [8]. We previously showed that the uncrosslinked glycans produced in mecillinam-treated cells are rapidly degraded by the lytic transglycosylase Slt to form soluble turnover products (anhydromuropeptides) [10]. Thus, in radiolabeled cells simultaneously inhibited for cell division and treated with mecillinam, the level of labeled turnover products produced provides a measure of RodA polymerization activity (Fig 3D). Using this assay, we found that both RodA(A234T) and PBP2(L61R) resulted in elevated PG turnover in mecillinam treated cells (Fig 3E and 3F). Similar assays were performed to monitor the effects of Rod system variants on aPBP activity using the beta-lactam cefsulodin. This antibiotic specifically inhibits the transpeptidase activity of aPBPs such that PG turnover in cefsulodin-treated cells provides a measure of aPBP PG polymerase activity [8,10]. Cefsulodin-induced PG turnover was found to be reduced in both RodA(A234T) and PBP2(L61R) containing cells (S3 Fig), indicating a reduction of aPBP polymerase activity. This reduction in activity most likely reflects an increased competition for precursors between aPBPs and the activated Rod system. Based on the radiolabeling results we conclude that the RodA(A234T) and PBP2(L61R) variants enhance Rod system function by promoting PG polymerization by RodA.

### PBP2(L61R) activates PG polymerization by RodA in purified RodA-PBP2 complexes

**Fig 5:**
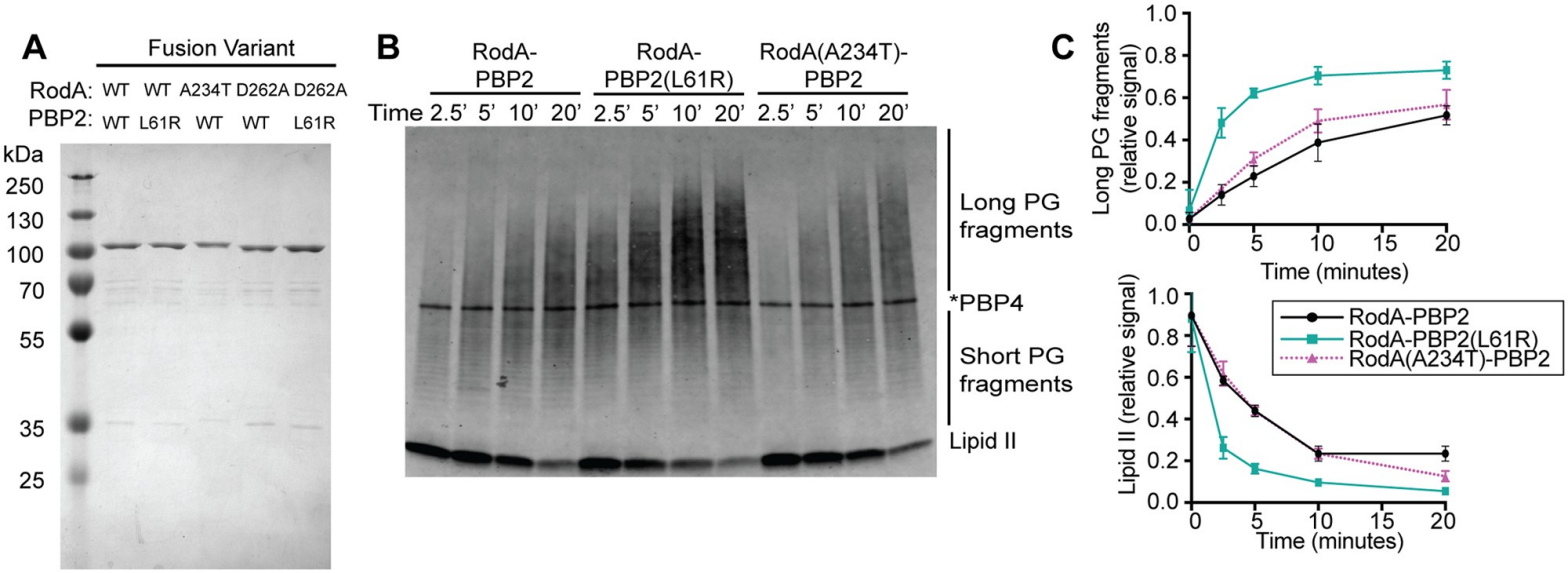
PBP2(L61R) stimulates glycosyltransferase activity of RodA. **A.** Purified FLAG-RodA-PBP2 and mutant derivatives were subjected to SDS-PAGE and stained using Coomassie Blue. The molecular weight of the fusion proteins is approximately 114 kDa. **B.** Blot detecting the peptidoglycan product produced by the RodA-PBP2 fusion constructs incubated with extracted *E*. *coli* Lipid II for the indicated length of time. The product was detected by biotin-D-lysine (BDL) labeling with *S*. *aureus* PBP4. Note that labeled PBP4 protein appears as a band in the middle of the blot. **C.** The accumulation of long PG fragments and depletion of lipid II during three independent replicates of glycosyltransferase time-courses were quantified using densitometry. Error bars represent standard deviation.

The in vivo labeling results suggest the attractive possibility that changes in PBP2 structure, either through its interaction with MreC or the L61R substitution, can be communicated to RodA to activate PG polymerization. We therefore wanted to test this potential RodA activation mechanism in vitro using purified RodA-PBP2 complexes. To simplify purification of the complexes, we generated a RodA-PBP2 fusion protein with the two components connected by a linker (GGGSx3). A similar SEDS-bPBP fusion had been shown to be functional for *Bacillus subtilis* sporulation [7]. Our construct was also active in vivo as it largely restored rod shape to Δ*pbpA-rodA* cells (S4 Fig). We therefore proceeded to purify a FLAG-tagged version of the wild-type fusion and fusions harboring either PBP2(L61R) or RodA(A234T). The fusions were produced in an *E*. *coli* expression strain lacking three of its four aPBP-type PG polymerases (PBP1b, PBP1c, and MtgA) to limit the potential for contaminating polymerase activity in the purified preparations. The resulting preparations were >90% pure with some observable lower molecular weight material (Fig 5A). Most of this material is derived from cleavage of the fusion within the linker, as the ~70 kDa band corresponds to the molecular weight of PBP2 and can be labeled with bocillin, while the ~40 kDa band corresponds to the molecular weight of FLAG-RodA and binds to an anti-FLAG antibody (S5 Fig).

We first compared the polymerase activity of RodA-PBP2(WT) with RodA-PBP2(L61R) and RodA(A234T)-PBP2. Purified lipid II substrate from *E*. *coli* was added to the fusions and the reactions terminated at various time points following initiation. The resulting products were then subjected to enzymatic labeling with biotin-D-lysine, separated using SDS-PAGE, transferred to a PVDF membrane, and detected with streptavidin conjugated to an infrared dye [32]. Mecillinam was included in the reactions to prevent glycan crosslinking by PBP2 so that polymer length could be determined without complications from crosslinking by PBP2. All fusions promoted the production of glycan polymers that increased in abundance and apparent length over time (Fig 5B and 5C). However, the RodA-PBP2(L61R) generated product more rapidly than RodA-PBP2(WT) and produced products that were longer (Fig 5B and 5C). The length and amount of PG produced by RodA(A234T)-PBP2 was not statistically different than the wild-type fusion (Fig 5B and 5C). Notably, the polymerase activity of all fusions was insensitive to moenomycin, an inhibitor that blocks aPBP-type PGT activity (S6 Fig). Also, the polymerase activity of fusions with PBP2(WT) and PBP2(L61R) was completely blocked by a D262A substitution in RodA (S6 Fig). An equivalent change was previously shown to inactivate the polymerase activity of *B*. *subtilis* RodA [4]. Therefore, the polymerase activity observed for the fusions is unlikely to be due to contaminating PBP1a, the only aPBP-type polymerase produced in the expression strain. We conclude that SEDS-bPBP complexes indeed form a functional PG synthase as proposed previously [4,8], and that changes in the bPBP can be communicated to the SEDS protein to stimulate its PG polymerase activity.

### PBP2(L61R) increases the number of functional Rod complexes per cell

Fluorescent protein fusions to MreB and other Rod system components in *E*. *coli* and *B*. *subtilis* form multiple dynamic foci dispersed throughout the cell cylinder. These foci have been observed to rotate in a processive manner around the long axis of the cell [8,33–35], and this motion is blocked by inhibitors of PG synthesis. Thus, the dynamic behavior of MreB and other Rod components is thought to be driven by the deposition of new PG material into the matrix with the speed of rotational movement reflecting the synthetic activity of the Rod complex.

**Fig 6:**
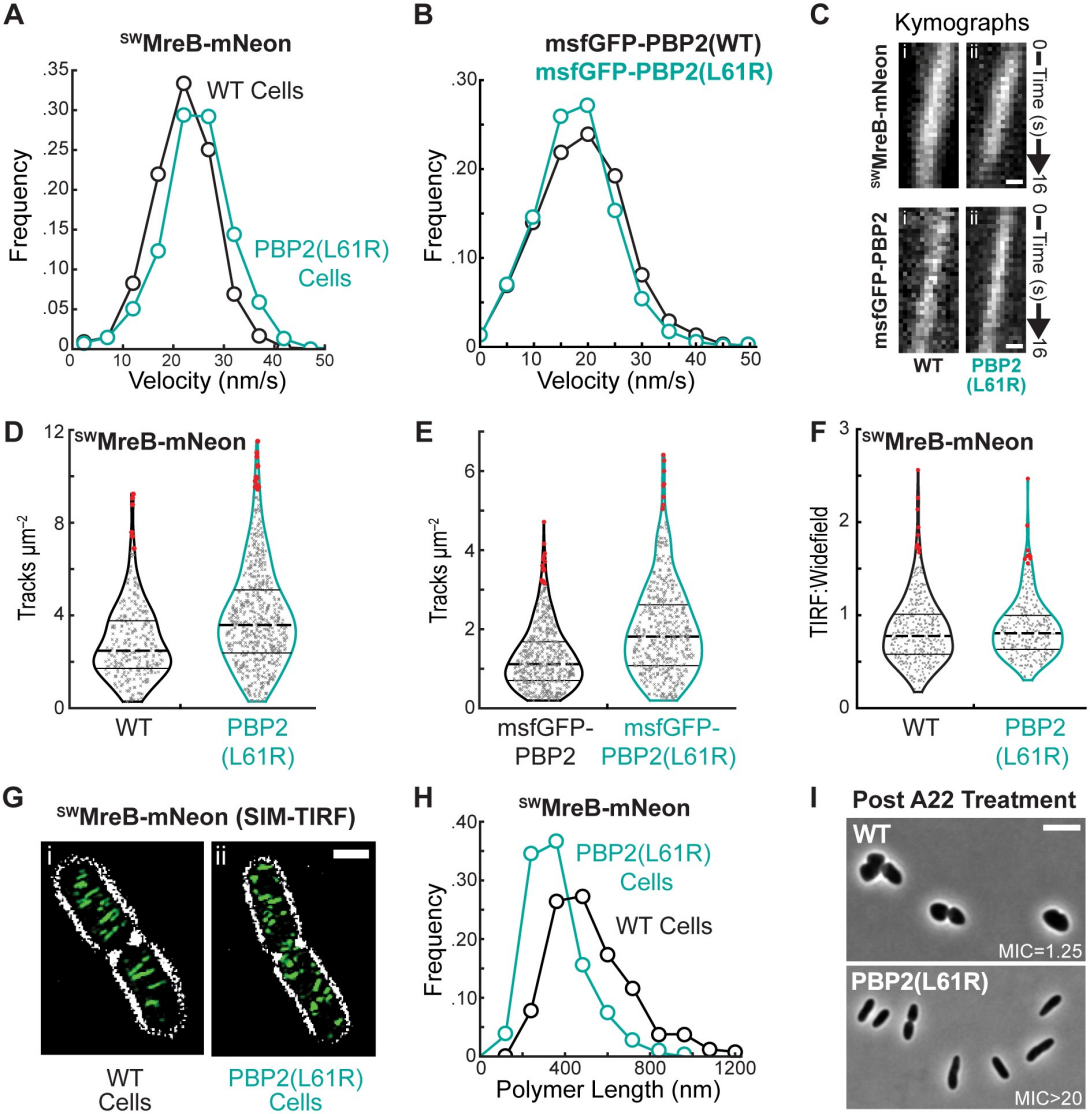
Rod system dynamics in PBP2(L61R) cells. **A.** Histograms of velocity measurements determined for individual traces of ^SW^MreB-mNeon (attλHC897) in wild-type (22±6 nm/s, n = 1467; black) and PBP2(L61R) cells [PR78] (25±7 nm/s, n = 949; turquoise). **B.** Histograms of velocity measurements determined for individual tracks of msfGFP-PBP2 (attλHC943) (18.9±8.1 nm/s, n = 2692; black) and msfGFP-PBP2(L61R) (attλPR128) (17.8±7.5 nm/s, n = 3440; turquoise) in Δ*pbpA* cells. Bin size, 5 nm/s. **C.** Kymographs of individual ^SW^MreB-mNeon (attλHC897) tracks in wild-type (i) and PBP2(L61R) (ii) cells are displayed atop kymographs of individual msfGFP-PBP2 (attλHC943, i) or msfGFP-PBP2(L61R) (attλPR128, ii) tracks in Δ*pbpA* cells. Scale bar, 250nm. **D.** Violin plots for the number of ^SW^MreB-mNeon tracks measured per cell area (um^2^) in wild-type (2.8±1.58, n = 609; black) and PBP2(L61R) cells (3.85±2.14, n = 300; turquoise) at 37°C. p<1e-10, Mann-Whitney U. The distribution of values along the x-axis capture the frequency of measurements along the y-axis. Lines designate quartiles with the dotted line indicating the mean value. Outliers are highlighted in red. **E.** Violin plots for the number of msfGFP-PBP2 (1.16±0.84, n = 581; black) and msfGFP-PBP2 (1.92±1.19, n = 517; turquoise) tracks measured per cell area (um^2^) in Δ*pbpA* cells at 30°C. As expected [36] there are less directionally moving rod complexes at lower temperatures. p<1e-29, Mann-Whitney U. **F.** Violin plots illustrating the distribution of normalized fluorescence measurements for ^SW^MreB-mNeon (attλHC897) expressed in wild-type (0.83±0.36, n = 397) and PBP2(L61R) cells (0.85±0.31, n = 321). The fluorescence intensity acquired under TIRF illumination for individual cells was integrated and divided by similar measurements taken under widefield illumination, providing an approximation for the relative abundance of surface-associated ^SW^MreB-mNeon. **G.** Representative SIM-TIRF micrographs of ^SW^MreB-mNeon integrated at the native locus in wild-type [JAB593] and PBP2(L61R) cells [JAB576]. The signal for ^SW^MreB-mNeon is pseudocolored green and overlaid a contrast-adjusted phase-contrast image. Scale bar, 1μm. **H.** Distributions of ^SW^MreB-mNeon polymer lengths in wild-type [JAB593] (520±190nm, n = 502; black) and PBP2(L62R) cells [JAB576] (360±130nm, n = 614; turquoise). Bin size, 120nm. p<1e-53, Mann-Whitney U. **I.** Representative phase-contrast micrographs of wild-type [MG1655] or PBP2(L61R) [PR78] cells after a 4hr treatment with 2 μg/mL A22, an MreB-inhibitor. The minimum inhibitory concentration (MIC) of A22 for each cell type is displayed in μg/mL.

To further understand the mechanism of Rod system activation by the PBP2(L61R) variant, we monitored its effect on the localization dynamics of MreB and PBP2 using total internal reflection fluorescence (TIRF) microscopy. An MreB sandwich fusion with mNeonGreen (^SW^MreB-mNeon) and an N-terminal monomeric superfolder-GFP fusion to PBP2 (msfGFP-PBP2) were used for the imaging. Both fusions were previously shown to be functional [8]. ^SW^MreB-mNeon foci displayed processive rotational movement in cells producing PBP2(WT) or PBP2(L61R) (S1 and S2 Movies). The speed of rotational movement was unchanged by the PBP2(L61R) variant (Fig 6A and 6C). Similarly, msfGFP-PBP2(WT) and msfGFP-PBP2(L61R) formed foci that moved around the cell long axis with almost identical velocities (S3 and S4 Movies, Fig 6B and 6C). Although the speed of particle motion was unchanged by the PBP2(L61R) variant in each case, the number of moving particles per cell appeared to increase in cells producing the altered PBP2. We therefore quantified the number of particle tracks per unit of cell surface area for each imaging experiment. Indeed, more directionally moving ^SW^MreB-mNeon foci were observed per cell area in the PBP2(L61R) producing cells versus those with PBP2(WT) (Fig 6D). Likewise, cells expressing msfGFP-PBP2(L61R) possessed a greater number of directionally moving foci than those producing msfGFP-PBP2(WT) (Fig 6E). These results suggest that PBP2(L61R) not only stimulates RodA polymerase activity, but also promotes the assembly of more active Rod complexes per cell.

One possible way in which the PBP2(L61R) variant could increase the number of active Rod complexes per cell is via enhancing the recruitment of MreB filaments to the membrane. To investigate this possibility, we measured the total ^SW^MreB-mNeon fluorescence per cell by widefield illumination and the fluorescence at the cell surface using TIRF illumination. We then calculated the TIRF/widefield ratio for each cell as a measure of MreB membrane recruitment. To ensure equivalent illumination of cells producing PBP2(WT) or PBP2(L61R), we introduced a cytoplasmic mCherry marker into one of the strains, mixed them, and performed the TIRF and widefield measurements on both strains simultaneously. Strain identity was then determined by the presence or absence of the mCherry marker (S7 Fig). Two sets of measurements were made, one with the marked strain being PBP2(WT) and the other with the PBP2(L61R) strain being marked. The analysis revealed no significant change in the TIRF/widefield ratio of ^SW^MreB-mNeon fluorescence between cells with either PBP2(WT) or PBP2(L61R) (Fig 6F), indicating that the total amount of MreB recruited to the membrane is not altered by PBP2(L61R).

The observation that the PBP2(L61R) variant increases the number of directionally moving ^SW^MreB-mNeon foci per cell without increasing the total amount of MreB at the membrane suggested that the altered synthase may be modulating MreB filament formation. To investigate this possibility, we imaged ^SW^MreB-mNeon using structured-illumination microscopy combined with TIRF illumination (SIM-TIRF). With this super-resolution method, clear filaments of ^SW^MreB-mNeon were visible that displayed a dynamic circumferential motion like the foci observed at lower resolution (Fig 6G, S5 Movie). Analysis of still images of cells with PBP2(WT) or PBP2(L61R) allowed us to measure the relative length of the fluorescent MreB filaments. Strikingly, the filaments observed in PBP2(L61R) cells were on average significantly shorter than those found in cells producing PBP2(WT) (Fig 6H). This observation suggests that changes in PBP2 affect MreB polymer formation and/or dynamics. Accordingly, similar to previously isolated PBP2 and RodA variants, cells producing PBP2(L61R) are resistant to the MreB antagonist A22 (Fig 6I), indicating that MreB polymers are more stable in these cells in addition to being altered in length. Overall, the cytological results are consistent with a model in which the activation status of the core PG synthase of the Rod system is communicated to MreB to modulate filament formation so that PG synthesis promoted by the activated enzymes is properly oriented.

## Discussion

**Fig 7:**
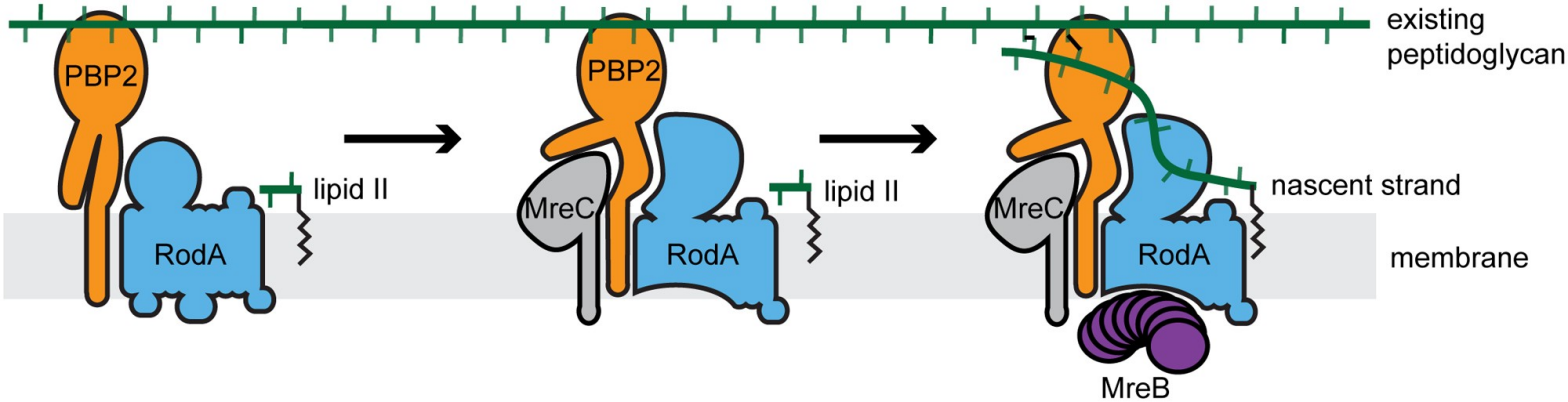
Proposed activation pathway governing peptidoglycan synthesis by the Rod System. In the absence of other factors, PBP2 and RodA are enzymatically inactive (left). In response to signals yet to be determined, MreC associates with PBP2, induced a conformational change, which in turn activates RodA (center). We propose that this activated complex has a higher affinity for MreB to promote MreB filament formation (right) to orient the PG produced by the activated synthase. For simplicity, MreD and RodZ are not shown but are also required for proper Rod system function, possibly by modulating the MreC-PBP2 interaction.

Cell shape determination in bacteria requires control of when and where new PG is made and incorporated into the existing matrix. It has been clear for some time that this spatiotemporal regulation is mediated by multiprotein complexes linked to cytoskeletal filaments [2]. However, an understanding of how the PG synthase enzymes within these machines are regulated has been lacking. It has also remained unclear how the polymerization state of the cytoskeletal filaments might affect the activation status of the synthases or vice versa. Our investigation of Rod system function suggests that its activity is governed by an activation pathway involving MreC, a component of the cell elongation machinery with heretofore unknown function, and the pedestal domain of PBP2 (Fig 7). The results also provide insight into how the synthetic activities of the PG polymerase and crosslinking enzyme within the complex are coordinated. Moreover, our results support a model in which activated PG synthesis enzymes influence MreB polymer formation, suggesting that MreB polymerization is not the only potential control point in Rod system activation. Finally, based on the similar nature of mutants activated for Rod system function to those bypassing normal regulation of the division machinery, we propose that the divisome is likely to be governed by an activation pathway controlling SEDS-bPBP synthases analogous to the one described here for Rod system regulation.

### A potential activation pathway controlling Rod system function

To gain insight into the regulation of the Rod system, we selected for suppressors of *mreC* missense mutants. Although the precise nature of the functional defect(s) caused by these mutations remains to be determined, they allowed us to identify two PBP2 variants that activate the Rod system. This activation both bypasses the need for some Rod system proteins, and hyperactivates the Rod system in otherwise wild-type cells. Characterization of the suppressor mutants combined with a recently solved structure of an MreC-PBP2 complex from *H*. *pylori* [27] supports a regulatory role for MreC in Rod system activation.

In the structure of the MreC-PBP2 complex, MreC was found to induce a significant conformational change in the membrane-proximal pedestal domain of PBP2, causing two of its subdomains to hinge open (Fig 2C) [27]. The amino-acid changes in PBP2 that suppress MreC defects mapped to the same region of the protein, suggesting that they may promote a conformation of PBP2 that mimics that induced by MreC. Biochemical and physiological results indicate that one of these altered PBP2 proteins, PBP2(L61R), not only suppresses MreC defects, it also stimulates Rod system activity in vivo and PG synthesis by RodA-PBP2 fusions in vitro. We infer from the combined set of results that the interaction between PBP2 and MreC is probably not just a scaffolding interaction as proposed previously [27], but also likely serves a regulatory role in Rod system function by shifting the RodA-PBP2 PG synthase into an activated conformation (Fig 7). Although a direct role for MreC in promoting RodA-PBP2 synthase activity remains to be tested, such an activation mechanism would ensure that the PG synthase is only highly active in the context of the assembled Rod complex thereby providing spatiotemporal control over its function.

In addition to suppressing the Rod system defect caused by missense alleles of *mreC*, the PBP2(L61R) variant also promoted viability and partially restored rod-shape to mutants deleted for *mreCD*, *rodZ*, and a triple *mreCD rodZ* deletion. However, the same PBP2 variant failed to suppress an *mreBCD* deletion, indicating that MreB is needed for Rod system function even when the core enzymes are abnormally activated. This MreB-requirement most likely reflects the important role of MreB filaments in promoting rod-shape by orienting the motion of the synthetic enzymes [12]. In this regard, the ability of PBP2(L61R) to promote partial Rod system function in the triple *mreCD rodZ* deletion is remarkable because it implies that MreB can interface directly with the RodA-PBP2 synthase. Thus, a cytoskeletal filament connected to a PG synthase complex appears to be the minimal functional unit of the Rod system in *E*. *coli*. The other components of the system are likely to be important for stabilizing the connection between RodA-PBP2 and MreB. However, because MreC, MreD, and RodZ are conserved along with RodA and PBP2 in ovoid and spherical bacteria lacking MreB, it seems unlikely that their sole function is to provide bridging interactions between the enzymes and MreB filaments. Instead, this conservation in combination with the suppression results with PBP2(L61R) suggests that like MreC, MreD and RodZ are probably also involved in promoting the activation of PG synthesis by RodA-PBP2, either directly or through an effect on the MreC-PBP2 interaction.

### MreB polymerization is modulated by the activation status of the RodA-PBP2 synthase

PBP2(L61R) cells were found to assemble more circumferentially moving MreB and PBP2 foci than PBP2(WT) cells. Additionally, super-resolution microscopy revealed that the MreB filaments formed at the membrane were shorter in the cells with the activated PBP2 variant. An increase in polymer number with a corresponding decrease in length is expected if polymer formation is stimulated without a change in the monomer supply. PBP2(L61R) was not found to alter the cellular MreB concentration or the total amount of MreB recruited to the membrane. Thus, the cytological results support a role for RodA-PBP2 activation in enhancing MreB polymerization, potentially by nucleating the formation of new polymers, either directly or through effects of the activated synthase on other Rod system components like RodZ [37]. Another connection between RodA-PBP2 activation and MreB polymerization comes from the observation that PBP2(L61R), and previously isolated PBP2 and RodA variants that are presumably also activated, confer resistance to the MreB antagonist A22 [24], indicating that the altered synthetic machinery is likely to be directly or indirectly stabilizing MreB polymers in addition to promoting their formation. Finally, MreB filament formation at the membrane has previously been shown to be dependent on the availability of the RodA-PBP2 substrate lipid II in *B*. *subtilis* [38]. Taken together, these observations support a model in which factors upstream of MreB polymerization are important control points in Rod system assembly and activation. Given the regulatory roles for MreC, MreD, and RodZ implied by the genetic results, an attractive possibility is that the membrane and periplasmic domains of these proteins function as sensors that promote PG synthesis by the Rod system in response to chemical and/or physical signals from the cell envelope such as PG crosslinking status, membrane curvature, or physical strain [39,40]. In this scenario, MreB filaments would be polymerized at or recruited to sites where synthesis is activated by the membrane-embedded components. Once recruited, MreB could then act as a rudder to steer cell wall insertion along the circumferential axis [12]. It is also possible that the activation process is initiated by MreB polymerization induced by a different set of stimuli. Importantly, the two possibilities are not mutually exclusive, and it may well be that multiple inputs into the formation of active Rod complexes contribute to the robustness of the system in promoting rod shape. A major challenge moving forward will be to determine the molecular nature of the signals to which the Rod system is responding to trigger its synthetic activity.

### Coupling of PG polymerization and crosslinking within the Rod system

Complexes between SEDS and bPBPs have been well described for the divisome (FtsW-PBP3) and sporulation (SpoVE-SpoVD) [6,7]. Therefore, following the discovery of PG polymerase activity for RodA, it was proposed that RodA-PBP2 and other SEDS-bPBP complexes form a functional PG synthase with both polymerase and crosslinking activity [4,8]. This possibility is supported by recent evolutionary coupling analyses and mutational studies indicating that a RodA-PBP2 complex formed through interactions between RodA and the pedestal domain of PBP2 is likely to be critical for Rod system function [9]. Here, we found that changes in the PBP2 pedestal domain can activate PG synthesis by RodA in vivo and stimulate the activity of RodA-PBP2 fusions in vitro. Together, these observations suggest that the RodA-PBP2 complex not only physically connects the two enzymes, but also serves as a regulatory conduit used to coordinate their activities. In this case, the genetic, biochemical, and structural data support a model in which conformational changes in the pedestal domain of PBP2 induced by MreC, likely in conjunction with other components of the system, are communicated to RodA to stimulate PG synthesis. This level of communication between the PGT and TP enzymes is attractive because it would provide a means to prevent RodA from robustly producing glycan strands without the ability to crosslink them. Otherwise, as revealed by experiments with the beta-lactam mecillinam, the production of uncrosslinked glycans by RodA when PBP2 is inactive results in a toxic futile cycle of glycan synthesis and degradation [10].

### A possible conserved regulatory mechanism governing PG synthesis by SEDS-bPBP synthases

Based on analogy with RodA-PBP2, FtsW-PBP3 has been proposed to be the core PG synthase of the divisome [4,8]. Recent biochemical studies from our laboratories indicate that FtsW indeed possesses PG polymerase activity and that this activity requires the formation of a complex with its cognate bPBP [16]. This finding is consistent with a required coupling between PG polymerase and crosslinking functions to prevent the formation of toxic uncrosslinked glycans. Genetic evidence in the literature also suggests that the FtsW-PBP3 complex is regulated by a mechanism analogous to that of RodA-PBP2. Several gain-of-function alleles in the genes encoding FtsW and PBP3 were previously isolated as suppressors of division inhibitor overproduction in *Caulobacter cresentus* and *E*. *coli* [13–15]. Notably, FtsW(A246T) was one of the suppressors of division inhibition identified in *C*. *cresentus* [15]. This residue change corresponds to A234T in *E*. *coli* RodA, the exact change that we and others have found to activate PG biogenesis by the Rod system and suppresses defects in MreC and RodZ [24]. Moreover, the amino acid substitutions in PBP3 that suppress division inhibition in *C*. *cresentus* map to the N-terminal domain not far from where we have found alterations in PBP2 that hyperactivate the Rod system [14]. Thus, the genetic evidence points towards PG biogenesis by the divisome being activated by the FtsW and PBP3 variants such that normal regulatory controls governing the activity of the complex can be bypassed. The similarity of these changes to those in RodA and PBP2 that activate the Rod system suggest that SEDS-bPBP complexes within morphogenic machines are likely to be regulated by similar and broadly conserved mechanisms. This activation step therefore represents an attractive target for small molecule inhibitors for use in antibiotic development.

## Methods

### Media, bacterial strains, and plasmids

All *E*. *coli* strains used in the reported experiments are derivatives of MG1655 [41]. Strains were grown in LB (1% tryptone, 0.5% yeast extract, 0.5% NaCl) or minimal M9 medium [42] supplemented with 0.2% casamino acids and 0.2% glucose (abbreviated M9 CAA glu). Unless otherwise indicated, antibiotics were used at 25 (chloramphenicol; Cm), 50 (kanamycin; Kan), 50 (ampicillin; Amp), or 5 (tetracycline; Tet) μg/mL. Growth conditions for microscopy experiments are described in the figure legends. Detailed information about strain and plasmid constructions can be found in S1 Text. All strains are listed in S3 Table and all plasmids are listed in S4 Table.

### Selection for suppressors of *mreC* point mutants

Overnight cultures of PR5 [*mreC(R292H)*] or PR30 [*mreC(G156D)*] were grown at 30°C in M9 CAA glu. Serial dilutions of these cultures were plated on both permissive conditions (M9 CAA glu agar at 30°C) and conditions that are non-permissive for the growth and survival of spherical cells (LB or LB supplemented with 1% sodium dodecyl sulfate (SDS) at 30°C or 37°C) [19]. After 24 hours of incubation, colonies that appeared on the LB (± SDS) plates were replica streaked on LB agar and LB agar supplemented with 10 μg/mL A22. We reasoned that suppressor mutants that have restored Rod system function would be sensitive to A22 (A22^S^), whereas mutants that had found an alternative means to survive on LB, such as overexpression of *ftsZ*, would be resistant to A22 (A22^R^). All A22^S^ isolates were visually screened to confirm restoration of rod cell shape using a Nikon Eclipse 50i microscope equipped with a 100x Ph3 DL 1.25 NA lens. We found that the A22^S^ isolates tended to have elongated cell shape consistent with at least a partial restoration of Rod system function. Note that although the *pbpA(L61R) mreC(R292H)* double mutant identified in our suppressor selection and screen is A22^S^, a *pbpA(L61R)* mutant in an otherwise Rod^+^ cell promotes A22^R^. We infer from this differential A22 sensitivity that the *pbpA(L61R)* allele can promote Rod system function when either MreB or MreC function is disrupted but not when both proteins are disabled.

Overnight liquid cultures of SDS^R^, A22^S^, rod-shaped isolates were grown in LB at 30°C, and genomic DNA was prepared using a Wizard Genomic DNA Purification Kit (Promega) and Genomic DNA Clean & Concentrator-10 Kit(Zymo Research). Two different methods were used for whole genome sequencing of suppressor strains. Some suppressors were prepared for sequencing using a modified Nextera library preparation strategy, as described by Baym *et al*. [43]. Other suppressors were prepared for sequencing using the NEBNext Ultra DNA Library Prep Kit for Illumina according to manufacturer’s instructions. DNA concentrations were determined using the Qubit dsDNA HS Assay Kit and sizes were determined using a High Sensitivity D1000 screen tape run on an Agilent 4200 TapeStation system. Sequencing was performed using a MiSeq Reagent Kit v3, with the Miseq System (Illumina). Reads were mapped using the CLC Genomics Workbench software (Qiagen). In each suppressor with a *pbpA* mutation that was sequenced, the alteration of *pbpA* was the only genomic change from the parental strain that was detected.

### Immunoblotting

Proteins were run on a 10% polyacrylamide gel and transferred to an activated PVDF membrane. The membrane was briefly rinsed, then blocked with 2% milk (w/v) in Tris-buffered saline, 0.1% Tween-20 (TBS-T) for 1 hour at room temperature. The membrane was then transferred to primary antibody solution, containing 0.2% milk (w/v), rabbit anti-MreB [19], rabbit anti-MreC (1:10,000 dilution), rabbit anti-FLAG (Sigma cat# F7245, 1 μg/mL), and/or mouse anti-RpoA (BioLegend clone 4RA2, 1:10000 dilution) in TBS-T, and incubated for 16 hours at 4°C. The membrane was rinsed quickly, then washed three times for ten minutes in TBS-T. The membrane was transferred to a solution of secondary antibodies (anti-rabbit 800CW and/or anti-mouse 680RD; Li-COR) in 0.1% milk for 1 hour at room temperature. After four ten-minute washes in TBS-T, the membrane was imaged using either a Li-COR ODESSEY Clx scanner or a ProteinSimple FluorChem R imager.

### Bocillin-binding assays

Bocillin-binding assays on membrane extracts were performed as described previously [8]. For bocillin binding assays of purified proteins, 8.3 μM of protein and 250 μM Bocillin-FL (ThermoFisher cat# B13233) were incubated for 30 minutes at room temperature. The protein was then combined with sample buffer and 10 pmol/lane was run on a 4–20% polyacrylamide gel. Bocillin gels were imaged using a Typhoon 9500 fluorescence imager (GE Healthcare) with excitation at 488 nm and emission at 530 nm.

### ^3^H-mDAP physiological radiolabeling

Peptidoglycan precursor levels, synthesis, and turnover were determined as described previously [8,10]. The results were analyzed using a two-way ANOVA, followed by Tukey’s multiple comparisons test.

### Protein expression

His-SUMO-FLAG tagged versions of RodA-GGGSx3-PBP2 wild-type and mutant fusions (encoded by pSS50, pSS51, pSS52, pSS60, and pSS62) were co-expressed with Ulp1 (encoded by pAM174) in an *E*. *coli* C43 derivative of BL21(DE3) with deletions in *ponB*, *pbpC*, *mtgA* (strain CAM333) [4]. CAM333/pAM174 cells with the desired pSS plasmid were grown at 37°C to an OD_600_ of 0.8 in 1L of Terrific Broth supplemented with 0.1% glucose and 2 mM MgCl_2_. IPTG was then added to 1 mM to induce expression of the fusion protein, and arabinose was added to 0.1% to induce expression of Ulp1. After induction overnight at 20°C, the cells were harvested by centrifugation. The cell pellets were resuspended in lysis buffer (50 mM HEPES pH 7.5, 150 mM NaCl, 20 mM MgCl_2_, 0.5 M DTT) and lysed by passage through a cell disruptor (Constant Systems Ltd.) twice at 25,000 psi. Membranes were collected by ultracentrifugation at 100,000g for 1 hour at 4°C. The membrane pellets were mechanically resuspended with a Teflon dounce homogenizer and solubilized in buffer containing 20 mM HEPES pH 7.0, 0.5 M NaCl, 20% glycerol, and 1% n-dodecyl-β-D-maltoside (DDM) for 2 hours at 4°C. Insoluble material was pelleted by ultracentrifugation at 100,000g for 1 hour at 4°C. The soluble fraction was removed and supplemented with 2 mM CaCl_2_ and applied to homemade M1 anti-FLAG antibody resin. The resin was washed with 25 mL of wash buffer (20 mM HEPES pH 7.0, 0.5 M NaCl, 20% glycerol, 2 mM CaCl_2_, 0.1% DDM). The FLAG-tagged constructs were eluted from the resin in 1 mL fractions with buffer containing 20 mM HEPES pH 7.0, 0.5 M NaCl, 20% glycerol, 0.1% DDM, 5 mM EDTA pH 8.0, and 0.2 mg/mL 3X FLAG peptide (Sigma). The purity of the sample was assessed by SDS-PAGE. The final yield for each of the different fusion constructs was approximately 1 mg per 1 L of culture.

A His-SUMO tagged version of the soluble domain of MreC (amino acids 45–367) was purified and used for antibody production. Lemo21(λDE3)/pPR57 cells were grown in LB supplemented with ampicillin and 25 g/mL chloramphenicol and grown at 37°C until the OD_600_ reached 0.4. Cells were then induced with 1 mM IPTG and grown for an additional 2 hours. Cells were pelleted and resuspended in buffer A (20 mM Tris-HCl (pH = 8.0), 300 mM NaCl, 0.5 mM DTT, 20% glycerol) containing 30 mM imidazole. cells were disrupted by passing them through a French pressure cell twice at 15,000 psi. Cell debris and membranes were pelleted by centrifugation at 100,000 x g for 30 minutes at 4°C. The resulting extract was mixed with pre-equilibrated QIAGEN Ni-NTA agarose beads, then transferred to a column. The column was washed sequentially with buffer A containing 30 mM, 50 mM, and 100 mM imidazole, then eluted in buffer A containing 300 mM imidazole. The eluate was digested with His-Ulp1 to cleave the His-SUMO tag, dialyzed in buffer A, then run through the Ni-NTA column to obtain pure, untagged MreC. Purified protein was sent to Covance Inc. for the production of rabbit polyclonal antibodies.

### Peptidoglycan glycosyltransferase activity assay

Purified proteins were concentrated to 10 μM using a 100 kDa MWCO Amicon Ultra Centrifugal Filter (Millipore). Extraction of *E*. *coli* Lipid II was performed as described previously [32]. Peptidoglycan glycosyltransferase activity was assayed as previously described [44]. Briefly, Lipid II dissolved in DMSO (μM) was incubated with each purified protein (1 μM) with 1X reaction buffer in a total volume of 10 μL for 20 minutes at room temperature, unless otherwise indicated. The reaction buffer contains 50 mM HEPES pH 7.0, 20 mM MgCl_2_, 20 mM CaCl_2_, 200 μM mecillinam, and 20% DMSO. Moenomycin dissolved in DMSO was used at a final concentration of 3 μM. Reactions were quenched by incubation at 95°C for 2 minutes. Biotinylation of the peptidoglycan product was subsequently performed by addition of 2 μL of 20 mM Biotin D-Lysine (BDL) and 1 μL of 50 μM *S*. *aureus* PBP4 (Kahne lab) and incubation at room temperature for 1 hour. The reaction was quenched with 13 μL of 2X SDS-loading buffer. 5 μL of the final reaction was loaded onto a 4–20% polyacrylamide gel and was run at 180V for 35 minutes. The peptidoglycan product was transferred onto an Immune-Blot PVDF membrane (BioRad). The Lipid II product, labeled with BDL, was detected by incubation with streptavidin-IRdye (Li-COR, 1:10,000 dilution).

To quantify blots of biotinylated products from glycosyltransferase assays, lane profiles were plotted using the Fiji gel analyzer tool [45]. Fragments larger than 48 kDa (the molecular weight of PBP4) were defined as long PG fragments. Fragments smaller than 48 kDa but larger than lipid II were defined as short PG fragments. The signal intensity from long PG fragments, short PG fragments, and lipid II were quantified and normalized to the total signal intensity in the lane. Results were analyzed using a two-way ANOVA, followed by Dunnett’s multiple comparisons test.

### Image acquisition and analysis

Growth conditions prior to phase-contrast microscopy are described in the figure legends. Where indicated, cells were fixed in 2.6% formaldehyde with 0.04% glutaraldehyde at room temperature for 1 h, followed by storage at 4°C for up to 3 days. Prior to imaging, cells were immobilized on 2% agarose pads containing the appropriate growth medium, and covered with #1.5 coverslips [46].

Phase-contrast microscopy was performed on a Nikon TE2000 microscope equipped with a 100x Plan Apo 1.4 NA objective, 0.90 NA condenser lens, and a CoolSNAP HQ2 monochrome camera (Photometrics). Images were acquired using software NIS Elements AR 3.2.

Single-molecule tracking of MreB and TIRF:widefield determinations were performed on a Nikon Eclipse Ti microscope equipped with a 100x Plan Apo 1.45 NA phase contrast objective and a Hamamatsu ORCA-Flash4.0 V2 (C11440-22CU) sCMOS camera. Fluorescence imaging was performed using a 488 nm excitation laser (Agilent Technologies) and an ET525/50 bandpass emission filter (Chroma Technology Corp). Image acquisition was performed using the Nikon Elements acquisition software. The microscope was maintained at 37°C using an environmental control chamber (World Precision Instruments).

All non-TIRF fluorescence imaging, as well as the single-molecule tracking of sfGFP-PBP2 variants were performed on a Nikon TiE instrument equipped with a 100x Plan Achromat 1.49 NA DIC objective, Andor Zyla 4.2 sCMOS camera, Ti-TIRF-EM Motorized Illuminator, a LUN-F laser launch with single fiber mode (488, 561, 640), Chroma TRF-EM 89901 Quad band set, Ti stage up kit, and Sutter Emission filter wheel. Environmental conditions were maintained using an Okolab stage top incubator chamber and a Bioptechs objective heater. Laser intensity was optimized to minimize phototoxicity. Acquisition software was NIS Elements 4.30. The purchase of this microscope was funded in part by grant S10 RR027344-01.

Sample preparation for fluorescence microscopy was performed as described previously [47]. Unless otherwise noted, cells were struck onto LB plates, inoculated in LB and grown overnight prior to back-dilution (1:500) into M9 minimal media on the day of imaging. Induction of *P*_*lac*_::^*SW*^*mreB-mNeon* (from attλHC897) was achieved with 100 μM IPTG throughout the duration of liquid growth. Imaging of *P*_*lac*_::*msfGFP-pbpA* (from attHKHC943) and *P*_*lac*_:*msfGFP-pbpA(L61R)* (from attHKPR128) required streaking onto M9 plates supplemented with 15 μM IPTG, followed by similar liquid growth. All conventional TIRF imaging was performed at 1s intervals for 1min duration with continuous illumination.

Analysis of phase-contrast images and widefield fluorescence was performed with Oufti [48] and MATLAB. Single-molecule tracking data was analyzed with the Fiji plugin TrackMate as described previously [8]. We discarded single-molecule trajectories if they consisted of < 5 consecutive frames and had a minimum displacement of < 70 nm. The number of tracks per cell was divided by the cell area determined by Oufti in order to normalize for cell shape differences. Cell areas were calculated as shown in S8 Fig.

### SIM-TIRF image acquisition and analysis

We acquired SIM-TIRF images on the DeltaVision OMX SR (GE Healthcare Life Sciences). Imaging was performed using a 60x 1.42 NA PSF objective with N = 1.522 immersion oil, and a 1.3x tube lens to provide additional magnification. Images were captured using a pro.edge sCMOS camera. The sample was maintained at 37°C using the built-in Environmental Control Module. Fluorescence imaging was conducted using a 488 nm excitation laser and a bandpass emission filter (528±2 nm center wavelength, 48±2 nm bandwidth). Imaging was performed at 37°C using ~20 ms acquisitions (9 per frame, ~200 ms total) at an interval of 1 s for 1–2 min duration. Images were acquired using the AcquireSR acquisition software (Applied Precision). For each frame of a SIM-TIRF time lapse, 9 images were taken (3 phases at 3 angles); these images were then reconstructed and the resulting time lapse was registered using the SI Reconstruction and Image Alignment functions in softWoRx (Applied Precision).

We determined ^SW^MreB-mNeon polymer lengths from SIM-TIRF snapshots by applying custom MatLab software similar to that previously described for an alternative super-resolution imaging technique [49]. Briefly, individual filaments deemed by eye to be entirely within the illumination area were rotated to a central axis and line-scanned (2-pixel width). Length was defined as the total number of contiguous pixels above local threshold (i.e. background + 50%) and reported in nm. Note, however, that the resolution of the imaging method is unlikely to provide an accurate measure of absolute filament length. Nevertheless, given that all SIM-TIRF images result from the same fluorescent fusion protein and were imaged under the same conditions and reconstructed with the same parameters, we believe that these measurements provide a valid comparison of relative filament length between strains. Also note that the average measured MreB filament filled ~½ of the total cell width in WT cells versus ~⅓ of the total cell width for cells with PBP2(L61R). Thus, a greater percentage of MreB filaments in WT cells had endpoints extending beyond the cell perimeter relative to cells with PBP2(L61R). Many long filaments in WT cells were therefore not measured such that our analysis is likely to have underestimated the length difference for MreB polymers between the two strains.

### TIRF:widefield measurements

Widefield illumination provides a depth-of-field of ~800 nm, approximating the entire fluorescent population within a cell. TIRF illumination provides a narrow depth-of-field (~200 nm) and approximates the membrane-associated population nearest the coverslip-sample interface. We assessed the relative abundance of the membrane-associated fraction of ^SW^MreB-mNeon within individual cells by calculating the ratio of the cumulative fluorescence intensity under TIRF and widefield. However, since TIRF intensity is highly affected by small changes in incident angle and z-focus, it is difficult to accurately compare separate TIRF:widefield datasets. Consequently we imaged both samples simultaneously. To differentiate the two strains, we expressed cytoplasmic mCherry (pAAY71) in either MG1655 attλHC897 or PR78 attλHC897 (S7 Fig). We used data from both imaging pairs for analysis **(**Fig 6F**)**.

## Supporting information

S1 Fig PBP2(L61R) cannot rescue Δ*mreBCD* cells expressing *mreCD* in trans. **A.** Overnight cultures of each strain grown in M9 CAA glu [MT4/pMS5, PR136/pMS5, PR139/pMS5] were diluted to OD_600_ = 0.05 in M9 CAA + 0.2% maltose + 100 μM IPTG and grown to OD_600_ = 0.2. Cells were then gently pelleted, resuspended and diluted in LB + 100 μM IPTG, and grown for three additional doublings (OD_600_ = 0.025 to OD_600_ = 0.2). At this point, cells were fixed, immobilized, and imaged using phase-contrast microscopy. All growth was performed at 30°C. Note that the *mreC* deletion is polar on *mreD* [18]. **B.** Overnight cultures of the above strains were serially diluted and spotted on either M9 CAA glu, LB, or LB + 100 μM IPTG. Plates were incubated at 30°C for either 40 h (M9) or 16 h (LB).
(TIF)

S2 Fig MreB and PBP2 levels are unaffected in the *pbpA(L61R)* mutant. **A.** Overnight cultures of each strain [PR132, PR78, PR150, PR151, TU230/pTB63] were diluted 1/200 and grown until the OD_600_ = 0.3, then labeled with Bocillin. Membrane fractions were isolated, and 15 μg of total protein was loaded in each lane of a 10% SDS-PAGE gel. Labeled protein was visualized using a Typhoon florescence scanner. **B.** Western blot detecting RpoA (red) and MreB (green). Each lane contains the indicated amount of total protein from exponential-phase (OD_600_ = 0.3) whole cell extracts of WT [PR132], *pbpA(L61R)* [PR78], and Δ*mreBCD*::*kan* [TU233/pTB63]. **C.** Western blot detecting RpoA (red) and MreB (green). Each lane contains the indicated amount of total protein from exponential-phase (OD_600_ = 0.3) whole cell extracts of WT [PR150], *rodA(A234T)* [PR151], and Δ*mreBCD*::*kan* [TU233/pTB63]. Note that PR132 is the parental strain of *pbpA(L61R)*, while PR150 is the parental strain of *rodA(A234T)*. The two strains have slightly different deletion/insertion mutations incorporating a resistance cassette into the *ybeM* pseudogene for use as a linked marker for strain constructions.
(TIF)

S3 Fig Increased PG synthesis in *pbpA* and *rodA* mutants is independent of aPBP activity. **A.** Labeling strains encoding PBP2(WT) or PBP2(L61R) at the native genomic locus [PR116(attHKHC859) and PR117(attHKHC859)] were pre-treated with 1.5 mM IPTG to induce SulA production, and 1 mM MTSES and/or 100 μg/mL cefsulodin, as indicated. Strains were then pulse-labeled with [3H]-mDAP, and peptidoglycan synthesis and turnover products (anhydroMurNAC-tripeptide and -pentapeptide) were measured. Results are the average of three independent experiments. Error bars represent the standard error of the mean. **B.** The same experiments and analysis as in (A) were performed using labeling strains encoding RodA(WT) or RodA(A234T) at the native genomic locus [PR146(attHKHC859) and PR147(attHKHC859)].
(TIF)

S4 Fig The RodA-PBP2 fusion is largely functional. **A.** Overnight cultures of cells deleted for the *pbpA-rodA* locus [HC558] harboring vectors producing the indicated native PBP2 and RodA proteins or RodA-PBP2 fusions from a P_lac_ regulated plasmid [pRY47, pHC857, pSS43] were diluted to OD_600_ = 0.005 in 3 mL of M9 medium supplemented with 0.2% casamino acids, 0.2% maltose, and 25 μM IPTG. When the OD_600_ reached 0.1–0.2, cells were fixed, immobilized and imaged using phase-contrast microscopy. Scale bar, 5 μm. **B.** Overnight cultures of the above strains were serially diluted and spotted on either M9 agar supplemented with 0.2% casamino acids and 0.2% maltose, or LB agar containing 50 μM IPTG.
(TIF)

S5 Fig A minor fraction of the RodA-PBP2 fusions are cleaved. **A.** Purified FLAG-RodA-PBP2 and mutant derivatives were run on an SDS polyacrylamide gel and stained with Coomassie blue, as in Fig 5A. **B.** Purified FLAG-RodA-PBP2 and mutant derivatives were stained with Bocillin-FL, separated by SDS-PAGE, and visualized using a Typhoon fluorescence scanner. **C.** Anti-FLAG western blot of purified FLAG-RodA-PBP2 and mutant derivatives. Note that the minor coomassie-stained bands in the purified preparations (panel A) correspond to Bocillin-labeled and/or FLAG-containing species in panels B and C. Thus, they are likely to represent minor cleavage products of the fusion as opposed to unrelated contaminants.
(TIF)

S6 Fig aPBP glycosyltransferase activity is not present in the purified RodA-PBP2 preparations. Blot detecting the peptidoglycan products produced by the RodA-PBP2 fusion constructs from the glycosyltransferase assays using *E*. *coli* Lipid II. The product was detected by biotin-D-lysine labeling with *S*. *aureus* PBP4. Glycosyltransferase activity was assessed in the presence and absence of moenomycin (moe). All reactions were analyzed after 20 min. SgtB, a moenomycin-sensitive glycosyltransferase purified from *S*. *aureus*, was used as a positive control. The introduction of a point mutation into RodA(D262A) disrupts the production of the polymerization product.
(TIF)

S7 Fig Representative images used for TIRF:widefield measurements. **A.** Representative micrographs of a mixed population containing MG1655(attλHC897) and PR78(attλHC897)/pAAY71(P_syn135_:mCherry). Images are presented as phase-contrast and fluorescence images overlaid with a contrast-adjusted phase-contrast image. Cytoplasmic mCherry (mCh) is pseudocolored red, while MreB-^SW^mNeon is pseudocolored green and labeled according to its illumination setting (TIRF, Widefield = WF). Scale bars, 1μm. **B.** Same as above, but with the mixed populations containing PR78(attλHC897) and MG1655(attλHC897)/pAAY71(P_syn135_:mCherry).
(TIF)

S8 Fig Calculations to determine the illuminated area of WT and PBP2(L61R) cells. **A-B.** Cartoons depicting the average cell length **A.** and width **B**. of WT (cyan) or PBP2(L61R) (magenta) cells that were imaged by conventional TIRF microscopy to determine the number of MreB-^SW^mNeon tracks cell^-1^. **C.** Given an estimated TIRF illumination depth of 200nm, we used the adjusted length and width dimensions to calculate the illuminated surface area for WT (3.01 um^2^) and PBP2(L61R) cells (3.38 um^2^). The illuminated surface area is displayed as a fraction of total surface area in parentheses.
(TIF)

S1 Movie Conventional TIRF microscopy of MreB-^SW^mNeon in WT cells [MG1655(attλHC857)]. (AVI)

S2 Movie Conventional TIRF microscopy of MreB-^SW^mNeon in PBP2(L61R) cells [PR78(attλHC857)]. (AVI)

S3 Movie Conventional TIRF microscopy of msfGFP-PBP2 in WT cells [TU230(attλHC943)]. (AVI)

S4 Movie Conventional TIRF microscopy of msfGFP-PBP2(L61R) in WT cells [TU230(attλPR128)]. (AVI)

S5 Movie SIM-TIRF microscopy of MreB-^SW^mNeon in WT cells [JAB593]. (AVI)

S6 Movie SIM-TIRF microscopy of MreB-^SW^mNeon in PBP2(L61R) cells [JAB576]. (AVI)

S1 Table Frequency of suppressor isolation from selections. (PDF)

S2 Table Growth rate and dimensions of cells with altered Rod system proteins. (PDF)

S3 Table Strains used in this study. (PDF)

S4 Table Plasmids used in this study. (PDF)

S1 Text Details for plasmid and strain constructions. (PDF)

## References

[1] Silver LL. Viable screening targets related to the bacterial cell wall. Ann N Y Acad Sci. 2013;1277: 29–53. 10.1111/nyas.12006 23278681

[2] Typas A, Banzhaf M, Gross CA, Vollmer W. From the regulation of peptidoglycan synthesis to bacterial growth and morphology. Nat Rev Microbiol. 2012;10: 123–136. 10.1038/nrmicro2677 22203377 PMC5433867

[3] Höltje JV. Growth of the stress-bearing and shape-maintaining murein sacculus of *Escherichia coli*. Microbiol Mol Biol Rev. 1998;62: 181–203. 9529891 10.1128/mmbr.62.1.181-203.1998 PMC98910

[4] Meeske AJ, Riley EP, Robins WP, Uehara T, Mekalanos JJ, Kahne D, et al SEDS proteins are a widespread family of bacterial cell wall polymerases. Nature. 2016 10.1038/nature19331 27525505 PMC5161649

[5] Henriques AO, Glaser P, Piggot PJ, Moran CP. Control of cell shape and elongation by the *rodA* gene in *Bacillus subtilis*. Mol Microbiol. 1998;28: 235–247. 9622350 10.1046/j.1365-2958.1998.00766.x

[6] Fraipont C, Alexeeva S, Wolf B, van der Ploeg R, Schloesser M, Blaauwen den T, et al The integral membrane FtsW protein and peptidoglycan synthase PBP3 form a subcomplex in *Escherichia coli*. Microbiology (Reading, Engl). 2011;157: 251–259. 10.1099/mic.0.040071-0 20847002

[7] Fay A, Meyer P, Dworkin J. Interactions between late-acting proteins required for peptidoglycan synthesis during sporulation. J Mol Biol. 2010;399: 547–561. 10.1016/j.jmb.2010.04.036 20417640 PMC2904850

[8] Cho H, Wivagg CN, Kapoor M, Barry Z, Rohs PDA, Suh H, et al Bacterial cell wall biogenesis is mediated by SEDS and PBP polymerase families functioning semi-autonomously. Nature Microbiology. 2016;1: 16172 10.1038/nmicrobiol.2016.172 27643381 PMC5030067

[9] Sjodt M, Brock K, Dobihal G, Rohs PDA, Green AG, Hopf TA, et al Structure of the peptidoglycan polymerase RodA resolved by evolutionary coupling analysis. Nature. 2018;556: 118–121. 10.1038/nature25985 29590088 PMC6035859

[10] Cho H, Uehara T, Bernhardt TG. Beta-lactam antibiotics induce a lethal malfunctioning of the bacterial cell wall synthesis machinery. Cell. 2014;159: 1300–1311. 10.1016/j.cell.2014.11.017 25480295 PMC4258230

[11] Alyahya SA, Alexander R, Costa T, Henriques AO, Emonet T, Jacobs-Wagner C. RodZ, a component of the bacterial core morphogenic apparatus. Proc Natl Acad Sci USA. 2009;106: 1239–1244. 10.1073/pnas.0810794106 19164570 PMC2633561

[12] Hussain S, Wivagg CN, Szwedziak P, Wong F, Schaefer K, Izoré T, et al MreB filaments align along greatest principal membrane curvature to orient cell wall synthesis. Elife. 2018;7: 1239 10.7554/eLife.32471 29469806 PMC5854468

[13] Du S, Pichoff S, Lutkenhaus J. FtsEX acts on FtsA to regulate divisome assembly and activity. Proc Natl Acad Sci USA. 2016;113: E5052–61. 10.1073/pnas.1606656113 27503875 PMC5003251

[14] Modell JW, Hopkins AC, Laub MT. A DNA damage checkpoint in Caulobacter crescentus inhibits cell division through a direct interaction with FtsW. Genes Dev. 2011;25: 1328–1343. 10.1101/gad.2038911 21685367 PMC3127433

[15] Modell JW, Kambara TK, Perchuk BS, Laub MT. A DNA damage-induced, SOS-independent checkpoint regulates cell division in Caulobacter crescentus. PLoS Biol. Public Library of Science; 2014;12: e1001977 10.1371/journal.pbio.1001977 25350732 PMC4211646

[16] Taguchi A, Welsh MA, Marmont LS, Lee W, Kahne D, Bernhardt TG, et al FtsW is a peptidoglycan polymerase that is activated by its cognate penicillin-binding protein. Preprint. 10.1101/358663 PMC6430707 30692671

[17] Kruse T, Bork-Jensen J, Gerdes K. The morphogenetic MreBCD proteins of *Escherichia coli* form an essential membrane-bound complex. Mol Microbiol. 2004;55: 78–89. 10.1111/j.1365-2958.2004.04367.x 15612918

[18] Bendezú FO, de Boer PAJ. Conditional lethality, division defects, membrane involution, and endocytosis in *mre* and *mrd* shape mutants of Escherichia coli. J Bacteriol. 2008;190: 1792–1811. 10.1128/JB.01322-07 17993535 PMC2258658

[19] Bendezú FO, Hale CA, Bernhardt TG, de Boer PAJ. RodZ (YfgA) is required for proper assembly of the MreB actin cytoskeleton and cell shape in E. coli. EMBO J. 2009;28: 193–204. 10.1038/emboj.2008.264 19078962 PMC2637328

[20] Shiomi D, Sakai M, Niki H. Determination of bacterial rod shape by a novel cytoskeletal membrane protein. EMBO J. 2008;27: 3081–3091. 10.1038/emboj.2008.234 19008860 PMC2599877

[21] Leaver M, Errington J. Roles for MreC and MreD proteins in helical growth of the cylindrical cell wall in *Bacillus subtilis*. Mol Microbiol. 2005;57: 1196–1209. 10.1111/j.1365-2958.2005.04736.x 16101995

[22] El Ghachi M, Matteï P-J, Ecobichon C, Martins A, Hoos S, Schmitt C, et al Characterization of the elongasome core PBP2: MreC complex of *Helicobacter pylori*. Mol Microbiol. 2011;82: 68–86. 10.1111/j.1365-2958.2011.07791.x 21801243

[23] Morgenstein RM, Bratton BP, Nguyen JP, Ouzounov N, Shaevitz JW, Gitai Z. RodZ links MreB to cell wall synthesis to mediate MreB rotation and robust morphogenesis. Proc Natl Acad Sci USA. 2015;112: 12510–12515. 10.1073/pnas.1509610112 26396257 PMC4603514

[24] Shiomi D, Toyoda A, Aizu T, Ejima F, Fujiyama A, Shini T, et al Mutations in cell elongation genes *mreB*, *mrdA* and *mrdB* suppress the shape defect of RodZ-deficient cells. Mol Microbiol. 2013;87: 1029–1044. 10.1111/mmi.12148 23301723 PMC3599482

[25] van den Ent F, Leaver M, Bendezu F, Errington J, de Boer P, Löwe J. Dimeric structure of the cell shape protein MreC and its functional implications. Mol Microbiol. 2006;62: 1631–1642. 17427287 10.1111/j.1365-2958.2006.05485.x

[26] Kelley LA, Sternberg MJE. Protein structure prediction on the Web: a case study using the Phyre server. Nat Protoc. 2009;4: 363–371. 10.1038/nprot.2009.2 19247286

[27] Contreras-Martel C, Martins A, Ecobichon C, Trindade DM, Matteï P-J, Hicham S, et al Molecular architecture of the PBP2-MreC core bacterial cell wall synthesis complex. Nat Commun. 2017;8: 776 10.1038/s41467-017-00783-2 28974686 PMC5626683

[28] Contreras-Martel C, Dahout-Gonzalez C, Martins ADS, Kotnik M, Dessen A. PBP active site flexibility as the key mechanism for beta-lactam resistance in pneumococci. J Mol Biol. 2009;387: 899–909. 10.1016/j.jmb.2009.02.024 19233207

[29] Powell AJ, Tomberg J, Deacon AM, Nicholas RA, Davies C. Crystal structures of penicillin-binding protein 2 from penicillin-susceptible and -resistant strains of *Neisseria gonorrhoeae* reveal an unexpectedly subtle mechanism for antibiotic resistance. J Biol Chem. 2009;284: 1202–1212. 10.1074/jbc.M805761200 18986991 PMC2613624

[30] Han S, Zaniewski RP, Marr ES, Lacey BM, Tomaras AP, Evdokimov A, et al Structural basis for effectiveness of siderophore-conjugated monocarbams against clinically relevant strains of *Pseudomonas aeruginosa*. Proc Natl Acad Sci USA. 2010;107: 22002–22007. 10.1073/pnas.1013092107 21135211 PMC3009787

[31] Dion M, Kapoor M, Sun Y, Wilson S, Ryan J, Vigouroux A, et al Cell Diameter in Bacillus subtilis is Determined by the Opposing Actions of Two Distinct Cell Wall Synthetic Systems. Preprint. 10.1101/392837 PMC6656618 31086310

[32] Qiao Y, Srisuknimit V, Rubino F, Schaefer K, Ruiz N, Walker S, et al Lipid II overproduction allows direct assay of transpeptidase inhibition by β-lactams. Nat Chem Biol. 2017;13: 793–798. 10.1038/nchembio.2388 28553948 PMC5478438

[33] Domínguez-Escobar J, Chastanet A, Crevenna AH, Fromion V, Wedlich-Söldner R, Carballido-López R. Processive movement of MreB-associated cell wall biosynthetic complexes in bacteria. Science. 2011;333: 225–228. 10.1126/science.1203466 21636744

[34] Garner EC, Bernard R, Wang W, Zhuang X, Rudner DZ, Mitchison T. Coupled, circumferential motions of the cell wall synthesis machinery and MreB filaments in B. subtilis. Science. 2011;333: 222–225. 10.1126/science.1203285 21636745 PMC3235694

[35] van Teeffelen S, Wang S, Furchtgott L, Huang KC, Wingreen NS, Shaevitz JW, et al The bacterial actin MreB rotates, and rotation depends on cell-wall assembly. Proc Natl Acad Sci USA. 2011;108: 15822–15827. 10.1073/pnas.1108999108 21903929 PMC3179079

[36] Billaudeau C, Chastanet A, Yao Z, Cornilleau C, Mirouze N, Fromion V, et al Contrasting mechanisms of growth in two model rod-shaped bacteria. Nat Commun. 2017;8: 15370 10.1038/ncomms15370 28589952 PMC5467245

[37] Morgenstein RM, Bratton BP, Shaevitz JW, Gitai Z. RodZ promotes MreB polymer formation and curvature localization to determine the cylindrical uniformity of *E*. *coli* shape. 2017 10.1101/226290 PMC6052060 30022070

[38] Schirner K, Eun Y-J, Dion M, Luo Y, Helmann JD, Garner EC, et al Lipid-linked cell wall precursors regulate membrane association of bacterial actin MreB. Nat Chem Biol. 2015;11: 38–45. 10.1038/nchembio.1689 25402772 PMC4270829

[39] Ursell TS, Nguyen J, Monds RD, Colavin A, Billings G, Ouzounov N, et al Rod-like bacterial shape is maintained by feedback between cell curvature and cytoskeletal localization. Proc Natl Acad Sci USA. 2014;111: E1025–34. 10.1073/pnas.1317174111 24550515 PMC3964057

[40] Wong F, Renner LD, Özbaykal G, Paulose J, Weibel DB, van Teeffelen S, et al Mechanical strain sensing implicated in cell shape recovery in *Escherichia coli*. Nature Microbiology. 2017;2: 17115 10.1038/nmicrobiol.2017.115 28737752 PMC5540194

[41] Guyer MS, Reed RR, Steitz JA, Low KB. Identification of a sex-factor-affinity site in *E*. *coli* as gamma delta. Cold Spring Harb Symp Quant Biol. 1981;45 Pt 1: 135–140. 6271456 10.1101/sqb.1981.045.01.022

[42] Miller JH. Experiments in Molecular Genetics. New York: Cold Spring Harbor Laboratory; 1972.

[43] Baym M, Kryazhimskiy S, Lieberman TD, Chung H, Desai MM, Kishony R. Inexpensive multiplexed library preparation for megabase-sized genomes. Green SJ, editor. PLoS ONE. 2015;10: e0128036 10.1371/journal.pone.0128036 26000737 PMC4441430

[44] Srisuknimit V, Qiao Y, Schaefer K, Kahne D, Walker S. Peptidoglycan Cross-Linking Preferences of *Staphylococcus aureus* Penicillin-Binding Proteins Have Implications for Treating MRSA Infections. Journal of the American Chemical Society. 2017;139: 9791–9794. 10.1021/jacs.7b04881 28691491 PMC5613940

[45] Schindelin J, Arganda-Carreras I, Frise E, Kaynig V, Longair M, Pietzsch T, et al Fiji: an open-source platform for biological-image analysis. Nature Methods. 2012;9: 676–682. 10.1038/nmeth.2019 22743772 PMC3855844

[46] Wang X, Montero Llopis P. Visualizing *Bacillus subtilis* During Vegetative Growth and Spore Formation. Methods Mol Biol. 2016;1431: 275–287. 10.1007/978-1-4939-3631-1_19 27283315

[47] Buss JA, Peters NT, Xiao J, Bernhardt TG. ZapA and ZapB form an FtsZ-independent structure at midcell. Mol Microbiol. 2017 10.1111/mmi.13655 28249098 PMC5426985

[48] Paintdakhi A, Parry B, Campos M, Irnov I, Elf J, Surovtsev I, et al Oufti: an integrated software package for high-accuracy, high-throughput quantitative microscopy analysis. Mol Microbiol. 2016;99: 767–777. 10.1111/mmi.13264 26538279 PMC4752901

[49] Buss J, Coltharp C, Xiao J. Super-resolution imaging of the bacterial division machinery. JoVE (Journal of Visualized Experiments). 2013 10.3791/50048 23380691 PMC3582665

