# Supplementary figures and images for: A central role for PBP2 in the activation of peptidoglycan polymerization by the bacterial cell elongation machinery
Source: PLoS Genet. 2018 Oct 18;14(10):e1007726. doi: 10.1371/journal.pgen.1007726 (PMC6207328; doi:10.1371/journal.pgen.1007726)

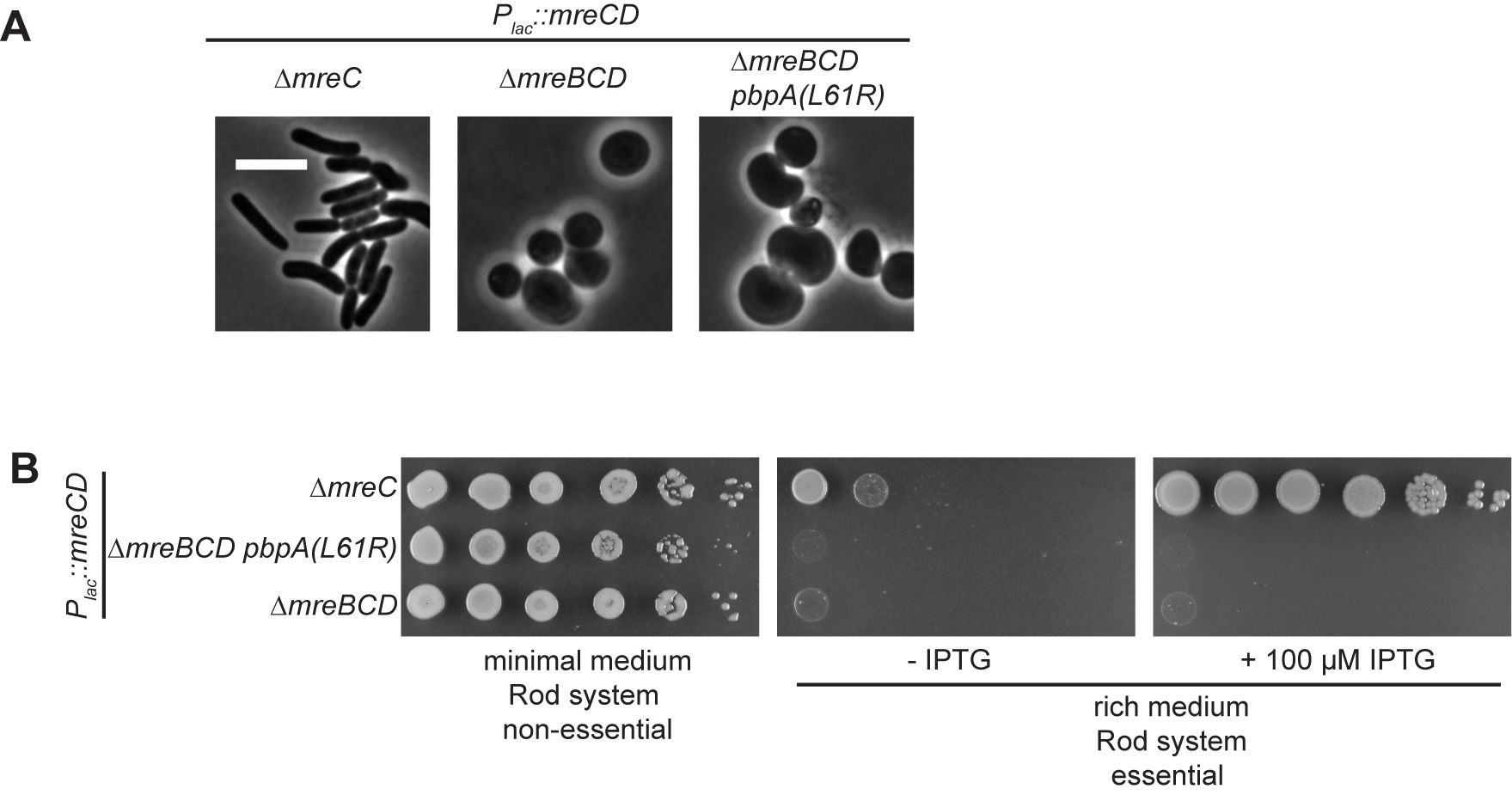

Supplement: S1 Fig — A. Overnight cultures of each strain grown in M9 CAA glu [MT4/pMS5, PR136/pMS5, PR139/pMS5] were diluted to OD600 = 0.05 in M9 CAA + 0.2% maltose + 100 μM IPTG and grown to OD600 = 0.2. Cells were then gently pelleted, resuspended and diluted in LB + 100 μM IPTG, and grown for three additional doublings (OD600 = 0.025 to OD600 = 0.2). At this point, cells were fixed, immobilized, and imaged using phase-contrast microscopy. All growth was performed at 30°C. Note that the mreC deletion is polar on mreD [18]. B. Overnight cultures of the above strains were serially diluted and spotted on either M9 CAA glu, LB, or LB + 100 μM IPTG. Plates were incubated at 30°C for either 40 h (M9) or 16 h (LB). (TIF) [file pgen.1007726.s001.tif]

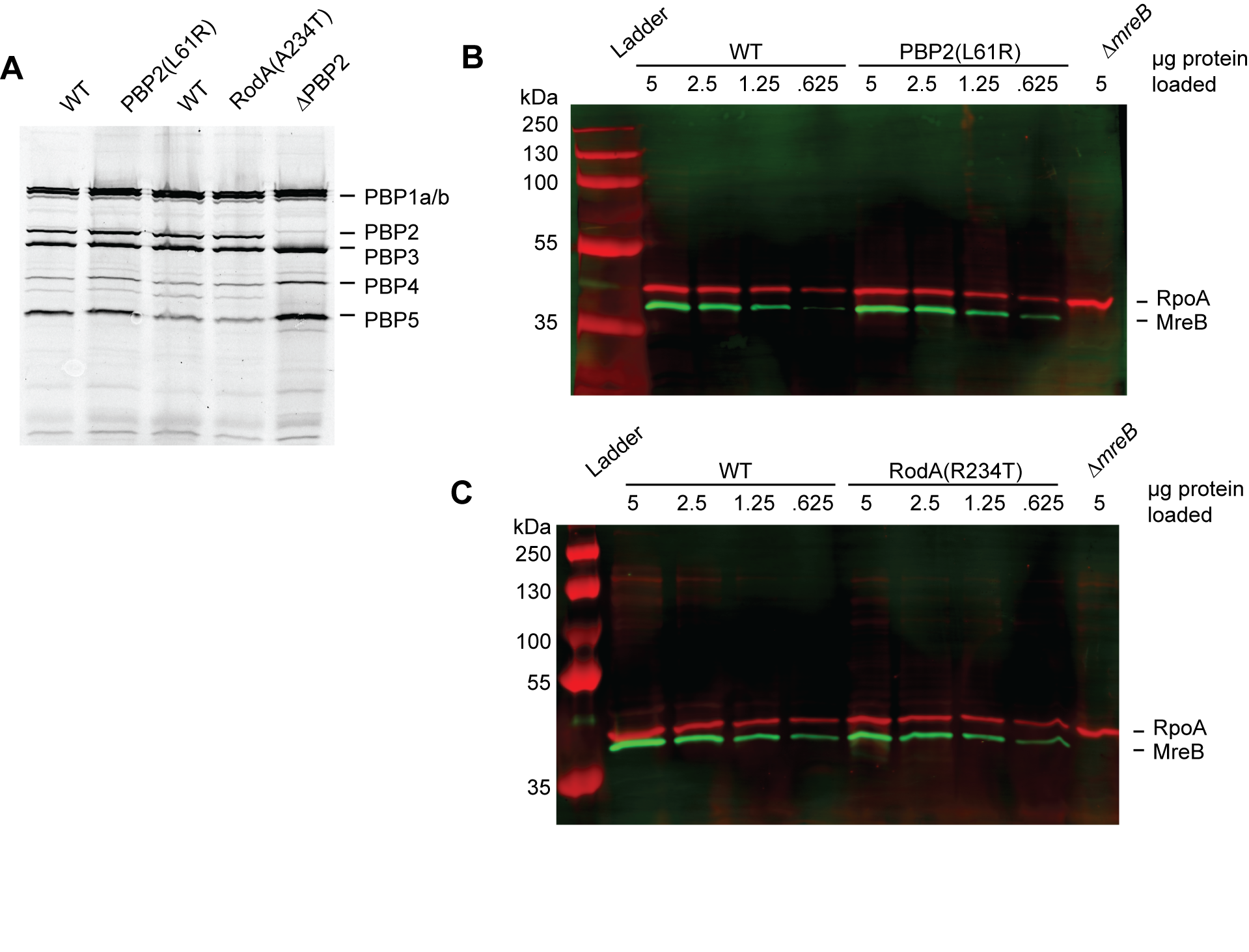

Supplement: S2 Fig — A. Overnight cultures of each strain [PR132, PR78, PR150, PR151, TU230/pTB63] were diluted 1/200 and grown until the OD600 = 0.3, then labeled with Bocillin. Membrane fractions were isolated, and 15 μg of total protein was loaded in each lane of a 10% SDS-PAGE gel. Labeled protein was visualized using a Typhoon florescence scanner. B. Western blot detecting RpoA (red) and MreB (green). Each lane contains the indicated amount of total protein from exponential-phase (OD600 = 0.3) whole cell extracts of WT [PR132], pbpA(L61R) [PR78], and ΔmreBCD::kan [TU233/pTB63]. C. Western blot detecting RpoA (red) and MreB (green). Each lane contains the indicated amount of total protein from exponential-phase (OD600 = 0.3) whole cell extracts of WT [PR150], rodA(A234T) [PR151], and ΔmreBCD::kan [TU233/pTB63]. Note that PR132 is the parental strain of pbpA(L61R), while PR150 is the parental strain of rodA(A234T). The two strains have slightly different deletion/insertion mutations incorporating a resistance cassette into the ybeM pseudogene for use as a linked marker for strain constructions. (TIF) [file pgen.1007726.s002.tif]

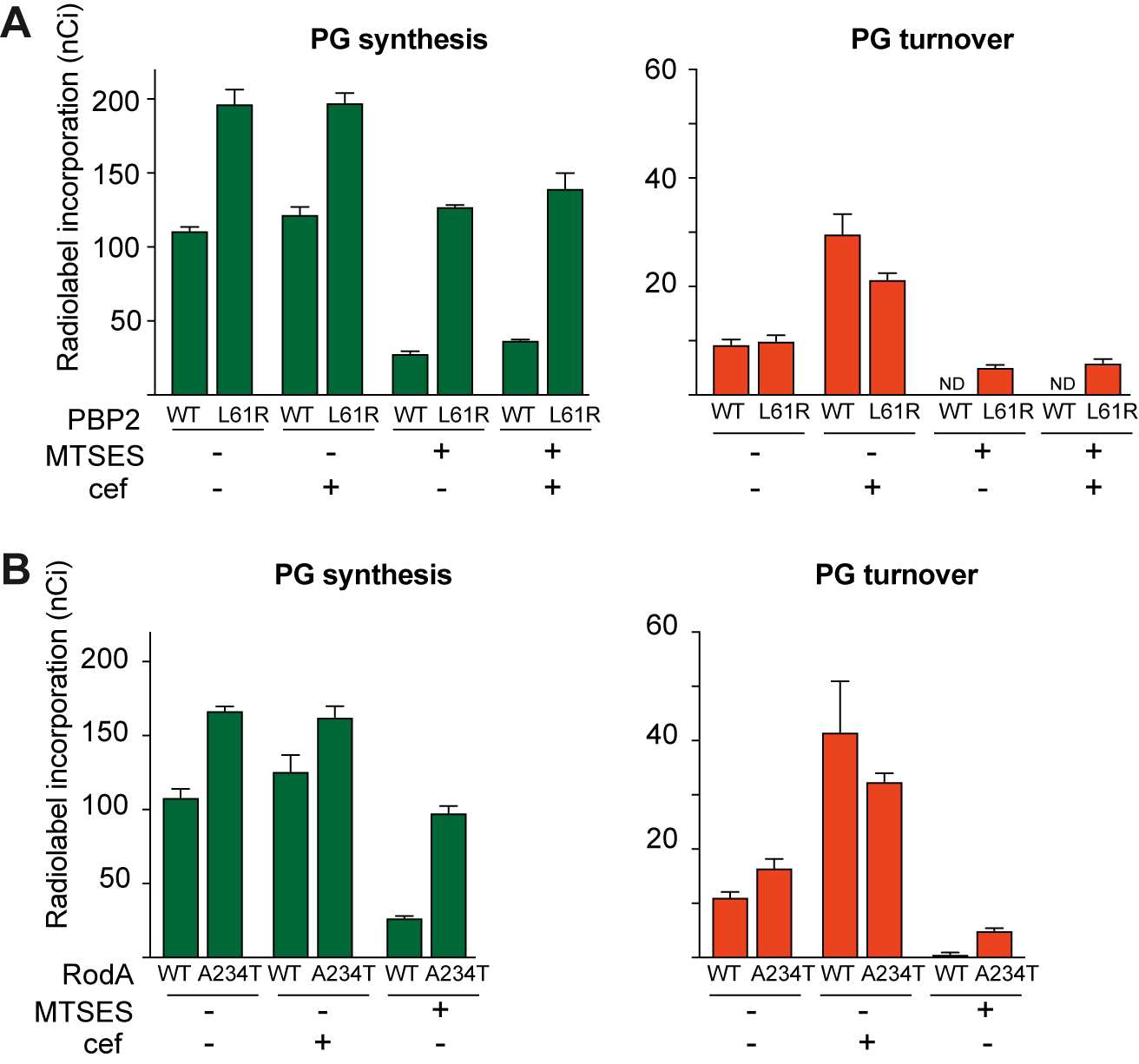

Supplement: S3 Fig — A. Labeling strains encoding PBP2(WT) or PBP2(L61R) at the native genomic locus [PR116(attHKHC859) and PR117(attHKHC859)] were pre-treated with 1.5 mM IPTG to induce SulA production, and 1 mM MTSES and/or 100 μg/mL cefsulodin, as indicated. Strains were then pulse-labeled with [3H]-mDAP, and peptidoglycan synthesis and turnover products (anhydroMurNAC-tripeptide and -pentapeptide) were measured. Results are the average of three independent experiments. Error bars represent the standard error of the mean. B. The same experiments and analysis as in (A) were performed using labeling strains encoding RodA(WT) or RodA(A234T) at the native genomic locus [PR146(attHKHC859) and PR147(attHKHC859)]. (TIF) [file pgen.1007726.s003.tif]

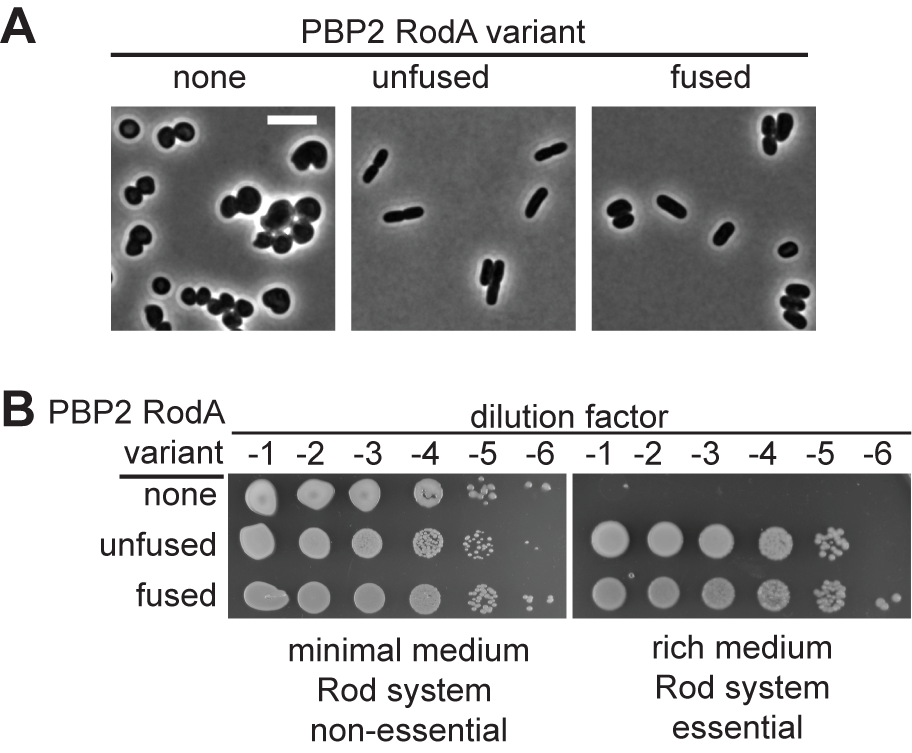

Supplement: S4 Fig — A. Overnight cultures of cells deleted for the pbpA-rodA locus [HC558] harboring vectors producing the indicated native PBP2 and RodA proteins or RodA-PBP2 fusions from a Plac regulated plasmid [pRY47, pHC857, pSS43] were diluted to OD600 = 0.005 in 3 mL of M9 medium supplemented with 0.2% casamino acids, 0.2% maltose, and 25 μM IPTG. When the OD600 reached 0.1–0.2, cells were fixed, immobilized and imaged using phase-contrast microscopy. Scale bar, 5 μm. B. Overnight cultures of the above strains were serially diluted and spotted on either M9 agar supplemented with 0.2% casamino acids and 0.2% maltose, or LB agar containing 50 μM IPTG. (TIF) [file pgen.1007726.s004.tif]

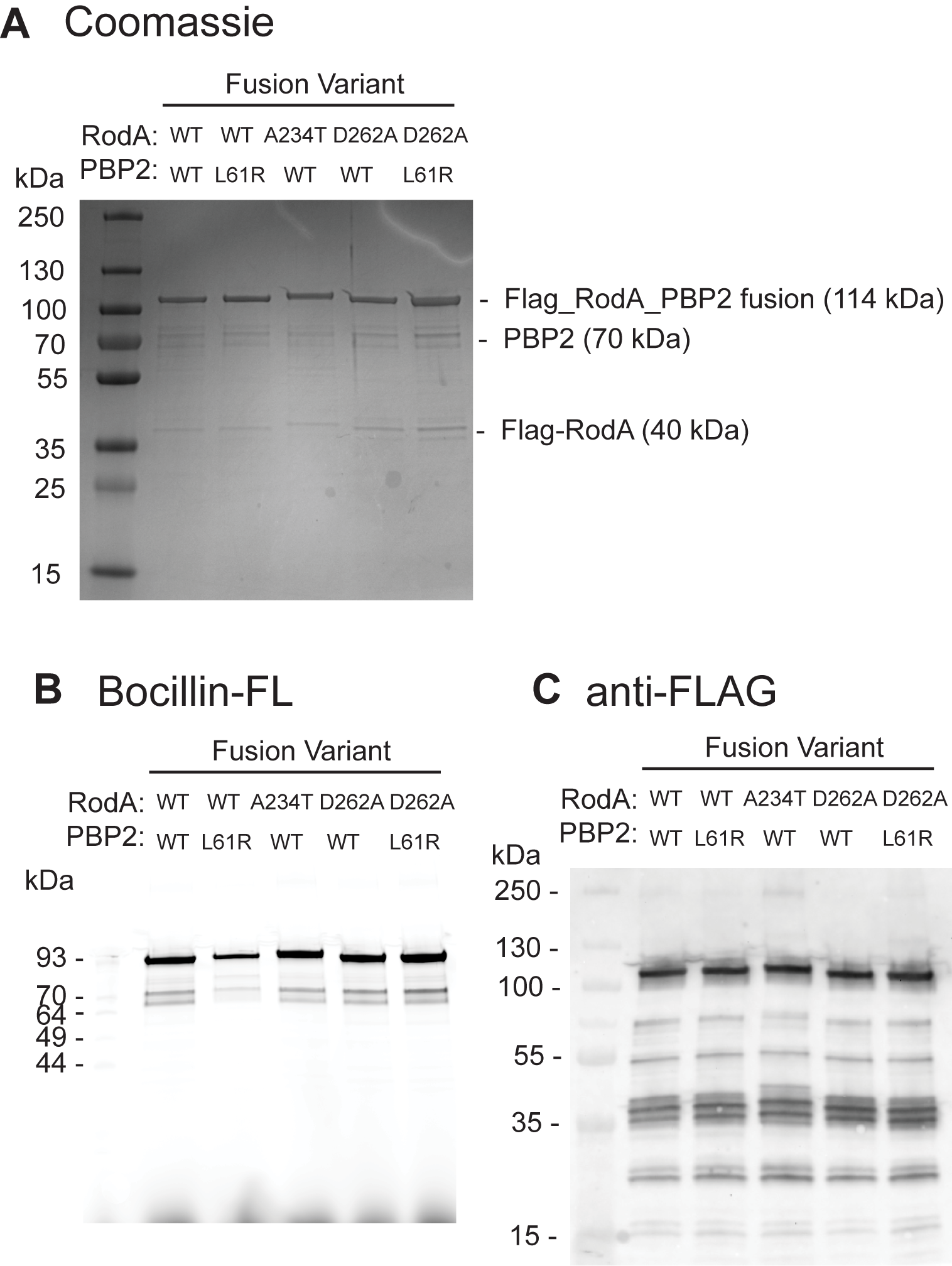

Supplement: S5 Fig — A. Purified FLAG-RodA-PBP2 and mutant derivatives were run on an SDS polyacrylamide gel and stained with Coomassie blue, as in Fig 5A. B. Purified FLAG-RodA-PBP2 and mutant derivatives were stained with Bocillin-FL, separated by SDS-PAGE, and visualized using a Typhoon fluorescence scanner. C. Anti-FLAG western blot of purified FLAG-RodA-PBP2 and mutant derivatives. Note that the minor coomassie-stained bands in the purified preparations (panel A) correspond to Bocillin-labeled and/or FLAG-containing species in panels B and C. Thus, they are likely to represent minor cleavage products of the fusion as opposed to unrelated contaminants. (TIF) [file pgen.1007726.s005.tif]

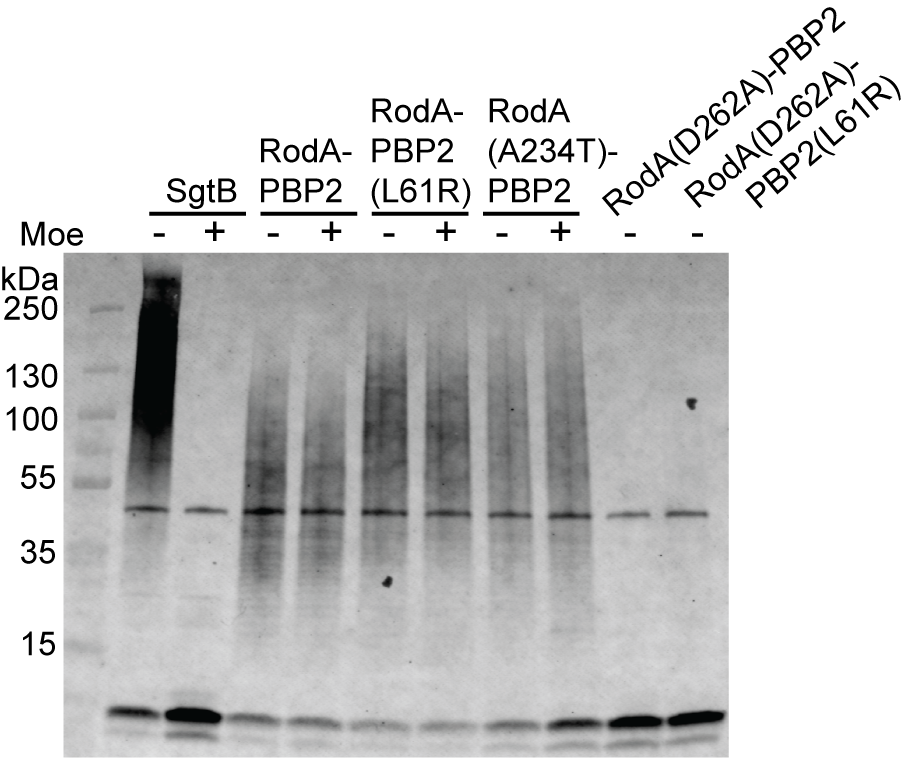

Supplement: S6 Fig — Blot detecting the peptidoglycan products produced by the RodA-PBP2 fusion constructs from the glycosyltransferase assays using E. coli Lipid II. The product was detected by biotin-D-lysine labeling with S. aureus PBP4. Glycosyltransferase activity was assessed in the presence and absence of moenomycin (moe). All reactions were analyzed after 20 min. SgtB, a moenomycin-sensitive glycosyltransferase purified from S. aureus, was used as a positive control. The introduction of a point mutation into RodA(D262A) disrupts the production of the polymerization product. (TIF) [file pgen.1007726.s006.tif]

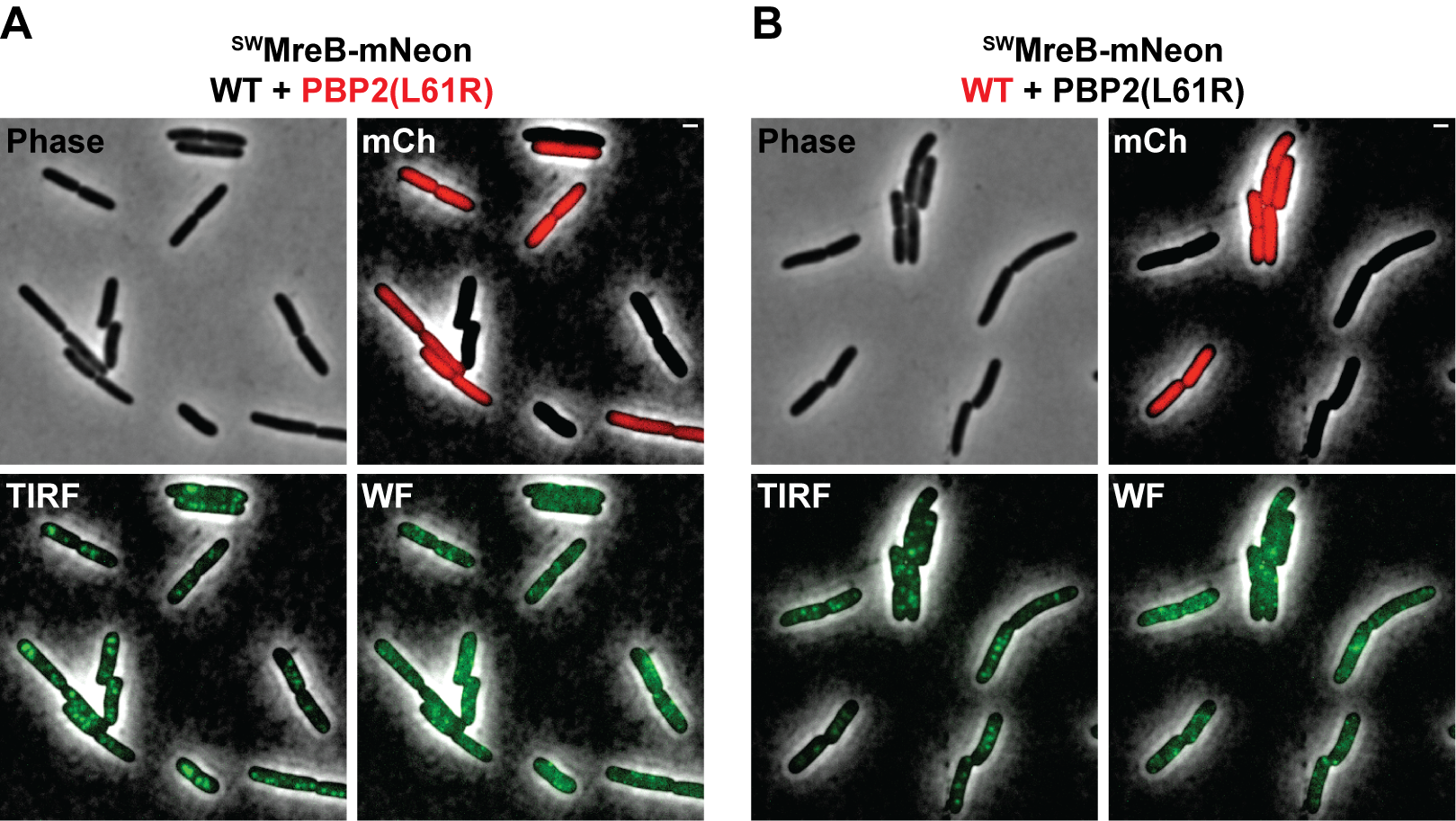

Supplement: S7 Fig — A. Representative micrographs of a mixed population containing MG1655(attλHC897) and PR78(attλHC897)/pAAY71(Psyn135:mCherry). Images are presented as phase-contrast and fluorescence images overlaid with a contrast-adjusted phase-contrast image. Cytoplasmic mCherry (mCh) is pseudocolored red, while MreB-SWmNeon is pseudocolored green and labeled according to its illumination setting (TIRF, Widefield = WF). Scale bars, 1μm. B. Same as above, but with the mixed populations containing PR78(attλHC897) and MG1655(attλHC897)/pAAY71(Psyn135:mCherry). (TIF) [file pgen.1007726.s007.tif]

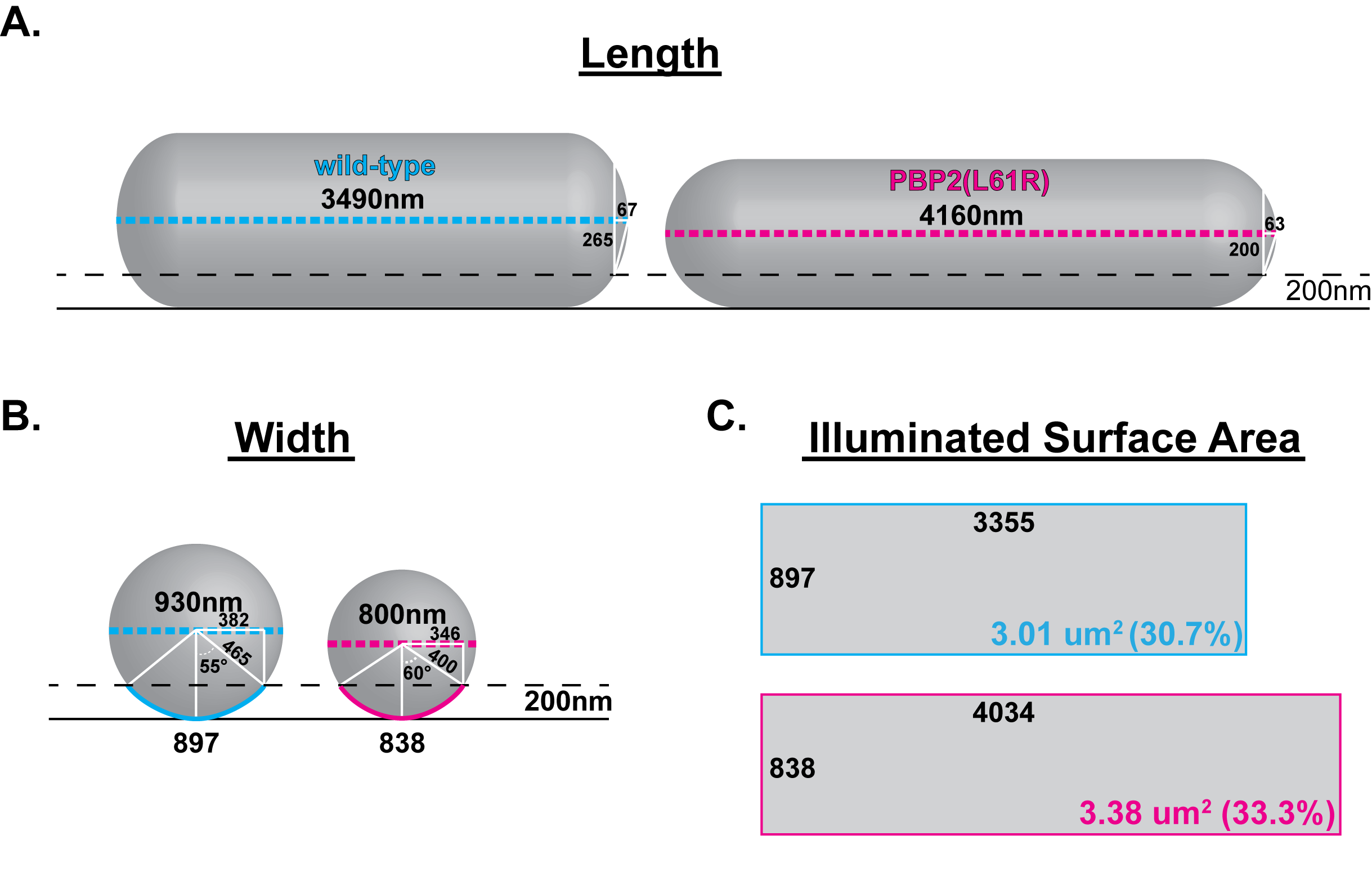

Supplement: S8 Fig — A-B. Cartoons depicting the average cell length A. and width B. of WT (cyan) or PBP2(L61R) (magenta) cells that were imaged by conventional TIRF microscopy to determine the number of MreB-SWmNeon tracks cell-1. C. Given an estimated TIRF illumination depth of 200nm, we used the adjusted length and width dimensions to calculate the illuminated surface area for WT (3.01 um2) and PBP2(L61R) cells (3.38 um2). The illuminated surface area is displayed as a fraction of total surface area in parentheses. (TIF) [file pgen.1007726.s008.tif]
