# Supplementary material for: A central role for PBP2 in the activation of peptidoglycan polymerization by the bacterial cell elongation machinery
Source: PLoS Genet. 2018 Oct 18;14(10):e1007726. doi: 10.1371/journal.pgen.1007726 (PMC6207328; doi:10.1371/journal.pgen.1007726)
Supplement: S1 Table — (PDF) [file pgen.1007726.s015.pdf]

S1 Table. Frequency of suppressor isolation from selections

Parental genotype	Selection condition	Frequency of suppressors	Fraction A22^S
mreC(R292H)	LB, 30 ℃	4.5×10^{-5}	74/133 (56%)
	LB, 37 ℃	6.8×10^{-5}	7/54 (13%)
	LB + SDS, 30 ℃	8.2×10^{-7}	36/38 (95%)
	LB + SDS, 37 ℃ [a]	8.2×10^{-6}	19/19 (100%)
mreC(G156D)	LB, 30 ℃	4.8×10^{-6}	11/21 (52%)
	LB + SDS, 30 ℃ [b]	1.3×10^{-7}	29/30 (97%)

[a] *pbpA(L61R)* isolated in this selection

[b] *pbpA(G156D)* isolated in this selection
