# Supplementary material for: A central role for PBP2 in the activation of peptidoglycan polymerization by the bacterial cell elongation machinery
Source: PLoS Genet. 2018 Oct 18;14(10):e1007726. doi: 10.1371/journal.pgen.1007726 (PMC6207328; doi:10.1371/journal.pgen.1007726)
Supplement: S2 Table — (PDF) [file pgen.1007726.s016.pdf]

S2 Table. Growth rate and dimensions of cells with altered Rod system proteins

Strain	Genotype	Doubling Time (min)		n	Cellular Dimensions[c] (μm)	
		LB[a]	M9[b]		length	width
MG1655	WT	43.3 ± 1.1	80.2 ± 1.4	1561	3.04 ± 0.06	1.03 ± 0.03
PR78	*ybeM1::FRT pbpA(L61R)*	42.9 ± 1.7	78.0 ± 3.0	1333	3.62 ± 0.07	0.88 ± 0.03
PR98	*ybeM1::FRT pbpA(L61R) yrdE::kan mreC(G156D)*	42.9 ± 0.9	76.8 ± 2.2	544	3.81 ± 0.97	0.97 ± 0.63
PR99	*ybeM1::FRT pbpA(L61R) yrdE::kan mreC(R292H)*	42.1 ± 0.9	84.4 ± 3.5	509	2.93 ± 0.63	1.09 ± 0.08
PR100	*ybeM1::FRT pbpA(L61R)* Δ*mreC::kan*	53.0 ± 5.7	121.7 ± 11.1	n/a	n/a	n/a
PR101	*ybeM1::cat*	41.7 ± 0.9	78.1 ± 4.0	504	3.02 ± 0.68	1.02 ± 0.08
MG1655 (attλHC857)	P_{Lac}:*mreB-*^{SW}*mNeon*			300	3.49 ± 0.69	0.93 ± 0.04
PR78 (attλHC857)	*ybeM1::FRT pbpA(L61R)* P_{Lac}:*mreB-*^{SW}*mNeon*			609	4.16 ± 0.90	0.80 ± 0.06
JAB593	*mreB-*^{SW}*mNeon* (at native locus)			837	3.15 ± 0.68	1.11 ± 0.07
JAB576	*ybeM1::FRT pbpA(L61R) mreB-*^{SW}*mNeon* (at native locus)			983	3.80 ± 0.88	1.06 ± 0.07
TU230 (attλHC943)	Δ*pbpA::kan* P_{native}:*sfGFP-pbpA*			577	3.17 ± 0.67	1.21 ± 0.10
TU230 (attλPR128)	Δ*pbpA::kan* P_{native}:*sfGFP-pbpA(L61R)*			517	3.65 ± 0.81	0.96 ± 0.08

[a]determined at 30°C, n=4

[b]determined at 30°C, n=3

[c]determined in M9 at 30°C
