# Supplementary material for: A central role for PBP2 in the activation of peptidoglycan polymerization by the bacterial cell elongation machinery
Source: PLoS Genet. 2018 Oct 18;14(10):e1007726. doi: 10.1371/journal.pgen.1007726 (PMC6207328; doi:10.1371/journal.pgen.1007726)
Supplement: S3 Table — (PDF) [file pgen.1007726.s017.pdf]

S3 Table. Strains used in this study

Strain	Genotype[a]	Source/Reference[b,c]
CAM333	*E. coli C43 ΔponB ΔpbpC ΔmtgA*	[9]
dH5α(λpir)	*F- hsdR17 deoR recA1 endA1 phoA supE44 thi-1 gyrA96 relA1 Δ(lacZYA-argF)U169 ∅80dlacZΔM15 λpir*	Laboratory stock
TU230	MG1655 *ΔpbpA::kan*	[5]
HC555	MG1655 *yrdE-kan*	P1(λRed) x MG1655
HC558	MG1655 *ΔpbpArodA::kan*	P1(λRed) x MG1655
JAB576	MG1655 *ybeM::frt mrdA(L61R) mreB'-mNeon-'mreB ΔyhdE::frt*	P1(HC583) [5] x PR78
JAB593	MG1655 *mreB'-mNeon-'mreB*	P1(HC583) [5] x MG1655
MG1655	*E. coli rph1 lvG rfb-50*	[10]
MT4	TB28 *ΔmreC::kan*	TB28 x P1(FB10) [11]
PM7	MG1655 *ΔybeM2::kan*	Piet de Boer, unpublished
PM11	MG1655 *ΔybeM2::kan rodA(A234T)*	Piet de Boer, unpublished
PR5	MG1655 *mreC(R292H) yrdE-kan*	P1(allelic exchange) x MG1655
PR30	MG1655 *mreC(G156D) yrdE-kan*	P1(allelic exchange) x MG1655
PR55	MG1655 *ΔybeM1::kan*	P1(λRed) x MG1655
PR68	MG1655 *ΔybeM1::kan pbpA(L61R)*	P1(allelic exchange) x MG1655
PR78	MG1655 *ΔybeM1::frt pbpA(L61R)*	PR68/pCP20
PR93	MG1655 *ΔybeM1::cat pbpA(L61R)*	P1(allelic exchange) x MG1655
PR98	MG1655 *ΔybeM1::frt pbpA(L61R) yrdE::kan mreC(G156D)*	PR78 x P1(PR30)
PR99	MG1655 *ΔybeM1::frt pbpA(L61R) yrdE::kan mreC(R292H)*	PR78 x P1(PR5)
PR100	MG1655 *ΔybeM1::frt pbpA(L61R) ΔmreC::kan*	PR78 x P1(MT4)
PR101	MG1655 *ΔybeM1::cat*	P1(λRed) x MG1655

Strain	Genotype[a]	Source/Reference[b,c]
PR115	MG1655 *ΔybeM1::cat pbpA(T52A)*	P1(suppressor strain) x MG1655
PR116	MG1655 *ΔlysA::frt ΔpbpC::frt ΔmtgA::frt ΔampD::frt mrcB(S247C) mrcA::frt ybeM1::cat*	HC533 [12] x P1(PR101)
PR117	PR116 *pbpA(L61R)*	HC533 [12] x P1(PR93)
PR124	PR164 *pbpA(T52A) mreC(R292H)*	PR115 x P1(PR5)
PR125	PR164 *pbpA(T52A) mreC(G156D)*	PR115 x P1(PR30)
PR127	PR164 *pbpA(L61R)*	PR93 x P1(HC555)
PR128	PR164 *pbpA(L61R) mreC(R292H)*	PR93 x P1(PR5)
PR129	PR164 *pbpA(L61R) mreC(G156D)*	PR93 x P1(PR30)
PR131	PR164 *pbpA(T52A)*	PR115 x P1(HC555)
PR132	MG1655 *ΔybeM1::frt*	PR101/pCP20
PR134	MG1655 *ΔrodZ::cat*	P1(λRed) x MG1655
PR136	PR132 *ΔmreBCD::kan*	PR132 x P1(FB30) [11]
PR137	PR132 *ΔmreCD::kan*	PR132 x P1(FB14) [11]
PR139	PR132 *pbpA(L61R) ΔmreBCD::kan*	PR178 x P1(FB30) [11]
PR140	PR132 *pbpA(L61R) ΔmreCD::kan*	PR178 x P1(FB14) [11]
PR142	PR132 *ΔrodZ::cat*	PR132 x P1(PR134)
PR143	PR143 *pbpA(L61R) ΔrodZ::cat*	PR78 x P1(PR134)
PR146	MG1655 *ΔlysA::frt ΔpbpC::frt ΔmtgA::frt ΔampD::frt mrcB(S247C) mrcA::frt ybeM2::kan*	HC533 [12] x P1(PM7)
PR147	PR146 *rodA(A234T)*	HC533 [12] x P1(PM11)
PR149	PR132 *pbpA(L61R) ΔmreCD::kan ΔrodZ::cat*	PR143 x P1(FB14) [11]
PR150	MG1655 *ΔybeM2::frt*	PM7/pCP20
PR151	PR150 *rodA(A234T)*	PM11/pCP20

Strain	Genotype[a]	Source/Reference[b,c]
PR152	PR150 *ΔmreBCD::kan*	PR150 x P1(FB30) [11]
PR153	PR150 *ΔmreCD::kan*	PR150 x P1(FB14) [11]
PR154	PR150 *ΔrodZ::cat*	PR150 x P1(PR142)
PR155	PR150 *rodA(A234T) ΔmreBCD::kan*	PR151 x P1(FB30) [11]
PR156	PR150 *rodA(A234T) ΔmreCD::kan*	PR151 x P1(FB14) [11]
PR157	PR150 *rodA(A234T) ΔrodZ::cat*	PR151 x P1(PR142)
PR158	MG1655 *ΔybeM2::frt yrdE-kan*	PR150 x P1(HC555)
PR159	PR158 *mreC(R292H)*	PR150 x P1(PR5)
PR160	PR158 *mreC(G156D)*	PR150 x P1(PR30)
PR161	PR158 *rodA(A234T)*	PR151 x P1(HC555)
PR162	PR158 *rodA(A234T) mreC(R292H)*	PR151 x P1(PR5)
PR163	PR158 *rodA(A234T) mreC(G156D)*	PR151 x P1(PR30)
PR164	MG1655 *ΔybeM1::cat yrdE-kan*	PR101 x P1(HC555)
PR165	PR164 *mreC(R292H)*	PR101 x P1(PR5)
PR166	PR164 *mreC(G156D)*	PR101 x P1(PR30)
Lemo21(λDE3)	*fhuA2 [lon] ompT gal (λDE3) [dcm] ∆hsdS/ pLemo*	[13]
SM10(λpir)	*KanR thi-1 thr leu tonA lacY supE recA::RP4-2-Tc::Mu attλ::pir*	[14]
TB10	MG1655 *λΔcro-bio nad::Tn10*	[15]
TB28	MG1655 *ΔlacIZYA::frt*	[16]

[a] The Kan^R cassette is flanked by *frt* sites for removal by FLP recombinase. An *frt* scar remains following removal of the cassette using FLP recombinase expressed from pCP20.

[b] Strain constructions by P1 transduction are described using the shorthand: P1(donor) x recipient. Transductants were selected on LB Kan, LB Tet, or LB Cm plates where appropriate. λRed indicates strains constructed by recombineering (see Experimental Procedures for details). Strains resulting from the removal of a drug resistance cassette using pCP20 are indicated as: Parental strain/pCP20.

[c] References are listed in **S1 Text**
