# Supplementary material for: A central role for PBP2 in the activation of peptidoglycan polymerization by the bacterial cell elongation machinery
Source: PLoS Genet. 2018 Oct 18;14(10):e1007726. doi: 10.1371/journal.pgen.1007726 (PMC6207328; doi:10.1371/journal.pgen.1007726)
Supplement: S4 Table — (PDF) [file pgen.1007726.s018.pdf]

S4 Table. Plasmids used in this study

Plasmid	Genotype[a]	Origin	Source or Reference[b]
pAAY71	*aacC1 Psyn135::mCherry*	pBBR/BHR	This study
pAM174	*cat araC* P_{BAD}*::Ulp1(403-621)*	pACYC/p15A	[9]
pCP20	*cat bla cI857* $P_{\lambda R}$*:FLP*	pSC101(ts)	[17]
pCX16	*aadA sdiA*	pSC101	[11]
pFB128	*aadA cI857(ts)* $P_{\lambda R}$*::mreD*	pSC101	[11]
pHC800	*cat* $lacI^q$ P_{tac}*::empty*	pBR/ColE1	[5]
pHC857	*cat* $lacI^q$ P_{lac}::nativeRBS-*pbpA-rodA*	pBR/ColE1	[5]
pHC859	*attHK022 tetA* $lacI^q$ P_{tac}*::sulA*	R6K	[18]
pHC897	*attλ cat* $lacI^q$ *Plac::mreB'-mNeonGreen-'mreB*	R6K	[12]
pHC943	*attHK022 tetAR* $lacI^q$ *Plac::msfgfp-GS-pbpA*	R6K	[12]
pKD13	*frt<bla>frt frt<kan>frt*	R6K	[1]
pKD3	*frt<cat>frt*	R6K	[1]
pKD46	*bla araC Para::γ-β-exo*	pSC101(ts)	[1]
pMS5	*cat* $lacI^q$ P_{lac}*::nativeRBS-mreCD*	pBR/ColE1	This study
pPR49	*cat* $lacI^q$ P_{tac}*::nativeRBS-mreC(R292H)-mreD*	pBR/ColE1	This study
pPR50	*cat* $lacI^q$ P_{tac}*::nativeRBS-mreC(G156D)-mreD*	pBR/ColE1	This study
pPR57	*bla* P_{T7}*:His6-SUMO-mreC(45-367)*	pBR/ColE1	This study
pPR84	*cat mobRP4 sacB mreC(R292H)mreD*	R6K	This study
pPR93	*cat mobRP4 sacB mreC(G156D)mreD*	R6K	This study
pPR101	*cat mobRP4 sacB rlmH pbpA(L61R)*	R6K	This study
pPR128	*attHK022 tetAR* $lacI^q$ *Plac::msfgfp-GS-pbpA(L61R)*	R6K	This study
pSS43	*cat* $lacI^q$ P_{lac}*::RodA'-GGGSx3-'PBP2*	pBR/ColE1	This study
pSS50	*bla* P_{T7}*:His6-SUMO-Flag-RodA'-GGGSx3-'PBP2*	pBR/ColE1	This study

Plasmid	Genotype[a]	Origin	Source or Reference[b]
pSS51	*bla* P_{T7}*:His6-SUMO-Flag-RodA'-GGGSx3-'PBP2(L61R)*	pBR/ColE1	This study
pSS52	*bla* P_{T7}*:His6-SUMO-Flag-RodA(A234T)'-GGGSx3-'PBP2*	pBR/ColE1	This study
pSS61	*bla* P_{T7}*:His6-SUMO-Flag-RodA(D262A)'-GGGSx3-'PBP2*	pBR/ColE1	This study
pSS62	*bla* P_{T7}*:His6-SUMO-Flag-RodA(D262A)'-GGGSx3-'PBP2(L61R)*	pBR/ColE1	This study
pTB63	*tetA* P_{native}*::ftsQAZ*	pSC101	[11]

[a] P_{ara}, $P_{\lambda R}$, P_{lac} and P_{tac} indicate the arabinose, λR, lactose, and tac promoters, respectively. Unless indicated, the ribosome binding site (RBS) used for all constructs is the strong RBS of phage T7 *Φ*10 gene.

[b] References are listed in **S1 Text**
