# Supplementary material for: A central role for PBP2 in the activation of peptidoglycan polymerization by the bacterial cell elongation machinery
Source: PLoS Genet. 2018 Oct 18;14(10):e1007726. doi: 10.1371/journal.pgen.1007726 (PMC6207328; doi:10.1371/journal.pgen.1007726)
Supplement: S1 Text — (PDF) [file pgen.1007726.s019.pdf]

A central role for PBP2 in the activation of peptidoglycan polymerization by the bacterial cell elongation machinery

Authors: Patricia D. A. Rohs[1], Jackson Buss[1¶], Sue I. Sim[1¶], Georgia R. Squyres[2], Veerasak Srisuknimit[3], Mandy Smith[1], Hongbaek Cho[4], Megan Sjodt[5], Andrew C. Kruse[5], Ethan C. Garner[2], Suzanne Walker[1], Daniel E. Kahne[3], and Thomas G. Bernhardt[1,6*]

[1]Department of Microbiology and Immunobiology, Harvard Medical School, Boston, Massachusetts, United States of America

[2]Department of Molecular and Cellular Biology, Harvard University, Cambridge, Massachusetts, United States of America

[3]Department of Chemistry and Chemical Biology, Harvard University, Cambridge, Massachusetts, United States of America

[4]Department of Biological Sciences, Sungkyunkwan University
Suwon, Gyeonggi, Korea

[5]Department of Biological Chemistry and Molecular Pharmacology, Harvard Medical School, Boston, Massachusetts, United States of America

[6]Howard Hughes Medical Institute, Boston, Massachusetts, United States of America

Short title: Regulation of the bacterial elongation system

*Corresponding author

E-mail: thomas_bernhardt@hms.harvard.edu (TGB)

¶These authors contributed equally to this work

Molecular biology

PCR was performed using Q5 polymerase (NEB) according to the manufacturer's instructions. Plasmid DNA and PCR fragments were purified using the Zyppy plasmid miniprep kit (Zymo Research) or the Qiaquick PCR purification kit (Qiagen), respectively. Sequencing reactions were carried out with an ABI3730xl DNA analyzer at the DNA Resource Core of Dana-Farber/Harvard Cancer Center (funded in part by NCI Cancer Center support grant 2P30CA006516-48).

Strain Constructions

A complete list of strains can be found in **S3 Table**.

HC555 [MG1655 *yrdE-kan*]*:* A Kan^R cassette was inserted in the intergenic space downstream of *yrdE* (genotype designated *yrdE-kan* in this paper) for use in transducing the *mre* locus with phage P1. The KanR cassette was amplified from pKD13 [1] using primers o1141 (TGGCGCTAATTTCGTGAATTGTGCGGCTTGTTGCAAATTAATTCCGGGGATCCGTCGACC) and o1142 (ATAATCAACAGCTAACATGTAAATAACCTTCAACACCGTGTGTAGGCTGGAGCTGCTTCG). The resulting PCR product was purified and electroporated into recombineering strain TB10 (using the same protocol as described for recombineering with DY330 [2]), and recombinants were selected at 30°C on LB agar supplemented with 25 µg/mL kanamycin. The *yrdE-kan* allele was moved from this strain into MG1655 by P1-

mediated transduction, generating strain HC555. The growth rate and cell dimensions of this strain are indistinguishable from wild type.

PR5 [MG1655 *mreC(R292H) yrdE-kan*]: A strain harboring the chromosomal *mreC(R292H)* mutation was constructed by allelic exchange, using a previously described protocol [3]. The *pir*-dependent suicide plasmid pPR84 *[sacB Cm^R]* was introduced into the recipient strain HC555/pTB63 *[yrdE-kan Tet^R]* by conjugative transfer from the donor strain SM10(λpir). Briefly, 5 mL of exponential-phase cultures (OD_{600} ≈ 0.3) of the donor and recipient strains were filtered onto the same 0.2 µm PES filter. This filter was placed cell-side-up on an LB agar plate and incubated for four hours at 37°C. Cells from the filter were then resuspended in 1 mL of LB, then plated on LB agar supplemented with chloramphenicol and tetracycline, and incubated at 30°C for 24 hours to select for exconjugants that contain pPR84 integrated into the chromosome via a single cross-over. Exconjugants were streaked on the same medium, and screened to identify isolates with spherical cell shape (indicating that the cross-over had occurred at the *mre* locus, resulting in *mreC(R292H)* expression). An exconjugant colony was resuspended in LB, serially diluted, plated on LB agar lacking NaCl and supplemented with 6% sucrose, and incubated at 30°C for 24 hours to select for recombinants that have lost the *sacB*-containing plasmid via a single cross-over. Sucrose-resistant colonies were replica-streaked on LB agar with and without chloramphenicol. Sucrose-resistant, chloramphenicol-sensitive isolates were screened for spherical cell morphology, indicating that *mreC(R292H)* had replaced the wild-type allele of *mreC* at the native chromosomal locus. This was confirmed by PCR followed by Sanger

sequencing. Strain PR5 was obtained by P1-mediated transduction of the genomic region near *yrdE-kan* (including *mre(R292H)*) from the primary isolate into MG1655. Transductants were selected on M9 agar supplemented with casamino acids, glucose, and kanamycin, screened for spherical cell shape, and confirmed by PCR and sequencing of *mreC.*

PR30 [MG1655 *mreC(G156D) yrdE-kan*]: A strain harboring the chromosomal *mreC(G156D)* mutation was constructed by allelic exchange using the suicide vector pPR93, following the same protocol as described above for PR5.

PR55 [MG1655 *ΔybeM1::kan*]: A Kan^R cassette was used to replace the *ybeM* pseudogene, so that this marker could be used to co-transduce the *mrd* locus. The Kan^R cassette was amplified from pKD13 [1] using primers o1237 (TCGTTGGCGAATTTTACGACTCTGACAGGAGGTGGCAATGATTCCGGGGATCCGTCGACC) and o1238 (AGCGCCGAGTAAAAAAACATCATAATAATTGCGGCGGCGCGTGTAGGCTGGAGCTGCTTCG). The resulting PCR product was purified and electroporated into recombineering strain TB10 (using the same protocol as described for recombineering with DY330 [2]), and recombinants were selected at 30°C on LB agar supplemented with 25 µg/mL kanamycin. The *ΔybeM::kan* allele was moved from this strain into MG1655 by P1-mediated transduction, generating strain PR55. The growth rate and cell dimensions of this strain are indistinguishable from wild type.

PR68 [MG1655 *ΔybeM1::kan pbpA(L61R)*]: A strain harboring the chromosomal *pbpA(L61R)* mutation was constructed by allelic exchange, using a previously described protocol [3]. The *pir*-dependent suicide plasmid pPR101 [*sacB* Cm^R] was introduced into the recipient strain PR55/pTB63 [*ΔybeM1::kan* / P_{native}*::ftsQAZ* Tet^R] by conjugative transfer from the donor strain SM10(λpir). Exconjugants that had integrated the plasmid into the genome via a single cross-over were selected on medium containing chloramphenicol and tetracycline. Exconjugants were then plated on sucrose to select for loss of the plasmid via a second recombination event. Suc^R Cm^S colonies were screened by PCR and sequencing for the presence of the *pbpA(L61R)* mutation. Strain PR68 was obtained by P1-mediated transduction of the genomic region near *ΔybeM::kan* (including *pbpA(L61R)*) from the primary isolate into MG1655.

PR101 [MG1655 *ΔybeM1::cat*]: A Cm^R cassette was used to replace the *ybeM* pseudogene, so that this marker could be used to co-transduce the *mrd* locus. The Cm^R cassette was amplified from pKD3 [1] using primers o1415 (TCGTTGGCGAATTTTACGACTCTGACAGGAGGTGGCAATGCATATGAATATCCTCCTTAG) and o1416 (AGCGCCGAGTAAAAAAACATCATAATAATTGCGGCGGCGCGTGTAGGCTGGAGCTGCTTC). These primers are designed such that the *ΔybeM1::cat* lesion is identical to the *ΔybeM1::kan* lesion in PR55, the only difference being the antibiotic resistance cassette. The resulting PCR product was purified and electroporated into recombineering strain TB10 (using the same protocol as described for recombineering with DY330 [2]), and recombinants were selected at 30°C on LB agar supplemented

with 25 µg/mL chloramphenicol. The *ΔybeM::cat* allele was moved from this strain into MG1655 by P1-mediated transduction, generating strain PR101. The growth rate and cell dimensions of this strain are indistinguishable from wild type **(S2 Table)**.

PR93 [MG1655 *ΔybeM1::cat pbpA(L61R)*]: The *ΔybeM1::cat* cassette was transferred from donor strain PR101 to recipient strain PR68 [MG1655 *ΔybeM1::kan pbpA(L61R)*] by P1-mediated transduction. Since *ybeM* and *pbpA* are closely linked, most transductants contained the wild-type *pbpA* sequence from donor strain PR101. PCR and sequencing were used to identify a rare Kan^S Cm^R transductant that retained the *pbpA(L61R)* sequence.

PR115 [MG1655 *ΔybeM1::cat pbpA(T52A)*]: This strain was constructed in a two-step procedure. First, the *ΔybeM1::cat* cassette from PR101 was transduced into a suppressor strain derived from PR30 [*mreC(G156D)*] that contains the spontaneous mutation *pbpA(T52A).* Although *ybeM* and *pbpA* are closely linked, all transductants retained the *pbpA(T52A)* mutation, because this mutation permits survival on LB. P1 lysates were prepared on this intermediate strain, and the *ΔybeM1::cat pbpA(T52A)* locus was co-transduced into MG1655, generating strain PR115. The presence of the *pbpA(T52A)* mutation was confirmed by PCR and sequencing.

PM7 [MG1655 Δ*ybeM2::kan*] was a gift from Dr. Piet de Boer. This strain contains a kanamycin resistance cassette in the *ybeM* locus. Since the exact junction points are different from those in PR55 [Δ*ybeM1::kan]*, the allele is designated Δ*ybeM2::kan.*

PM11 [MG1655 Δ*ybeM2::kan rodA(A234T)*] contains a *rodA(A234T)* mutation in the PM7 genetic background. This strain was a gift from Dr. Piet de Boer.

PR134 [MG1655 Δ*rodZ::cat*]: A Cm^R cassette was used to replace the region between the 2nd codon and 7th codon from the stop codon of *rodZ,* as described previously [2,4]. The Cm^R cassette was amplified from pKD3 [1] using primers o1953 (CTCCCGCGTTACCCGTCTGTTACTGCGCCGGTGATTGTTCGTGTAGGCTGGAGCTGCTTC) and o1954 (CGGCATCTCAATTCTCATTTAAACGTACCTGCAGCGAATGCATATGAATATCCTCCTTAG). The resulting PCR product was purified and electroporated into MG1655/pKD46 as described previously [1], and recombinants were selected at 42°C on M9 agar supplemented with casamino acids, glucose, and 25 µg/mL chloramphenicol. PR134 was made by P1 transduction of Δ*rodZ::cat* from this intermediate strain into an MG1655 recipient strain.

HC558 [MG1655 Δ*pbpArodA::kan*]: A Kan^R cassette was used to replace the region between the 2nd codon of *pbpA* and 5th codon from the stop codon of *rodA,* as described previously [2]. The Kan^R cassette was amplified from pKD13 [1] using primers o1094 (TGAGTGATAAGGGAGCTTTGAGTAGAAAACGCAGCGGATGATTCCGGGGATCCGTCGACC) and o1095 (CCACTGCTTACGCATTGCGCACCTCTTACACGCTTTTCGATGTAGGCTGGAGCTGC

TTCG). The resulting PCR product was purified and electroporated into TB10/pCX16, using the same protocol as described for recombineering with DY330 [2]), and recombinants were selected at 30°C on LB agar supplemented with 25 µg/mL kanamycin. HC558/pRY47, HC558/pHC857, and HC558/pSS43 were made by P1-mediated transduction of Δ*pbpArodA::kan* from this intermediate strain into MG1655 containing the corresponding plasmid.

Plasmid constructions

A complete list of plasmids can be found in **S4 Table**.

pMS5 [*colE1 cat lacI^q P_lac::nativeRBS-mreCD*]: The insert (*XbaI-nativeRBS-mreC-mreD-HindIII*) was amplified using MG1655 genomic template DNA and primers o882 (GTCA*TCTAGA*CTGCCTGGTCTGATACGAGAATACGCATAACTTATG) and o905 (GTCA*AAGCTT*TTATTGCACTGCAAACTGCTGACGG). This insert was cloned into pRY47 [5] using the XbaI and HindIII restriction sites.

pPR49 [*colE1 cat lacI^q P_tac::nativeRBS-mreC(R292H)mreD*]: Primers o882, o918 (CTGCATCAG**ATG**TTCATTAGCAACACGATGC), o919 (GCTAATGAA**CAT**CTGATGCAGATGATGCCGC), and o905 were used to amplify MG1655 genomic DNA and introduce the R292H mutation into *mreC* using overlap-extension PCR. The product was PCR purified, digested with XbaI/HindIII, and cloned into similarly digested pHC800 [5].

pPR50 [*colE1 cat lacI^q P_tac::nativeRBS-mreC(G156D)mreD*]: Primers o882, o914 (GACCAACAAC**ATC**TTTGTCGCTGATGACCGGC), o915 (GCGACAAA**GAT**GTTGTTGGTCAGGTGGTGG), and o905 were used to amplify MG1655 genomic DNA and introduce the G156D mutation into *mreC* using overlap-extension PCR. The product was PCR purified, digested with XbaI/HindIII, and cloned into similarly digested pHC800 [5].

pPR57 [*colE1 bla P_{T7}:His6-SUMO-mreC(45-367)*]: Primers o883 (GTCA*AAGCTT*CTATTGCCCTCCCGGCGCAC) and o920 (ATTGGT*GGATCC*GCCGTCAGTCCTTTCTACTTTGTTTCC) were used to amplify the insert (BamHI-mreC(45-367)-HindIII) from MG1655 genomic DNA. This insert was cut with BamHI/HindIII and ligated into similarly digested pTD68 [6].

pPR84 [*cat mobRP4 sacB mreC(R292H)mreD*]: Primers o1157 (GTCA*GAGCTC*CTGCCTGGTCTGATACGAG) and o1158 (GTCA*TCTAGA*TTATTGCACTGCAAACTGCTGACGG) were used to amplify the insert (SacI-mreC(R292H)-mreD-XbaI) from pPR49. This insert was cut with SacI/XbaI and ligated into similarly digested pDS132 [3].

pPR93 [*cat mobRP4 sacB mreC(G156D)mreD*]: Primers o1157 and o1158 were used to amplify the insert (SacI-mreC(G156D)-mreD-XbaI) from pPR50. This insert was cut with SacI/XbaI and ligated into similarly digested pDS132 [3].

pPR101 [*cat mobRP4 sacB rlmH pbpA(L61R)*]: Primers o1285 (GTCA*GAGCTC*CATCCGCTGGTTCGCGTGCTGG) and o1286 (GTCA*TCTAGA*TCCCCATATCGTAGGCCACCTG) were used to amplify the insert (a segment of genomic DNA encompassing a 3' fragment of *rlmH* and the 5' half of *pbpA(L61R),* flanked by SacI and XbaI restriction sites) from a suppressor mutant derived from PR5, containing the spontaneous mutation *pbpA(L61R)*. This insert was cut with SacI/XbaI and ligated into similarly digested pDS132 [3].

pPR128 [*attHK022 tetAR lacIq Plac::msfgfp-GS-pbpA(L61R)*]: *pbpA(L61R)* was PCR amplified from PR68 gDNA using primers o264 (GCTAAAGCTTTTTATTCGGATTATCCGTCATG) and o1041 (GCTA*GGATCC*AAACTACAGAACTCTTTTCGCGACTATACG). The resulting PCR product was digested with BamHI and HindIII restriction enzymes and cloned into pHC943, which was pre-digested with the same enzymes [8].

pSS43 [*colE1 cat lacI^q P$_{lac}$::RodA'-GGGSx3-'PBP2*] was generated in two steps. First, the insert containing RodA was amplified from MG1655 genomic DNA as a template with primers oSS37 (TCGACAAGCTTTTACACGCTTTTCGACAACATTTTCCTGTGG) and oSS59 (GTTTAACTTTAAGAAGGAGATATACCATGACGGATAATCCGAATAAAAAAACATTCTGGG). The resulting PCR product was then assembled with XbaI/HindIII-digested pRY47 *[colE1 cat lacI^q P$_{lac}$::empty]* using the isothermal assembly procedure [7]. This intermediate plasmid was amplified using primers oSS62

(CCGCAGCGGAGGACCATTAAGCTTGTCACCGATACGCGAGCGAACGTGAAGCGA
CTGCTG) and oSS75
(AGAACCGCCACCGGAGCCACCGCCGCTACCGCCACCCACGCTTTTCGACAACATT
TTCCT) to create the vector for isothermal assembly with an insert containing
GGGSx3-'PBP2, amplified from MG1655 genomic DNA with primers oSS61
(CTCGCGTATCGGTGACAAGCTTAATGGTCCTCCGCTGCGGCAACCGCTGGATTTT
CCGCA) and oSS74
(GGTGGCGGTAGCGGCGGTGGCTCCGGTGGCGGTTCTAAACTACAGAACTCTTTTC
GCGAC).

pSS50 [*colE1 bla P_{T7}:His6-SUMO-FLAG-RodA'-GGGSx3-'PBP2*] was generated in a two-piece isothermal assembly reaction with an insert containing RodA'-GGGSx3-'PBP2, which was amplified from pSS43 [*colE1 cat lacIq P_{lac}::RodA'-GGGSx3-'PBP2*] with oligonucleotide primers oSS82
(GGGTCATCCACGGATAATCCGAATAAAAAAACATTCTGGGATAAAGTCCATCTCGAT
CCC) and oSS84
(GCAGCCGGATCCCCTTCCTGCAGTCACCCGGGCTTAATGGTCCTCCGCTGCGGC
AACCGC), and pAM172 *[colE1 bla P_{T7}::His6-SUMO-FLAG-RodA]* [4], which was amplified with oligonucleotide primers oSS83
(TCCCAGAATGTTTTTTTATTCGGATTATCCGTGGATGACCCCCCAGGGCCTTGAAAC
AAC) and oSS85
(AATCCAGCGGTTGCCGCAGCGGAGGACCATTAAGCCCGGGTGACTGCAGGAAGG
GGATCC).

pSS51 [*colE1 bla P_{T7}:His6-SUMO-FLAG-RodA'-GGGSx3-'PBP2(L61R)*] was generated in a two-piece isothermal assembly reaction with an insert containing PBP2(L61R), which was amplified from PR68 gDNA with oligonucleotide primers oSS74 and oSS84, and pSS50, which was amplified with oligonucleotide primers oSS75 and oSS85.

pSS52 [*colE1 bla P_{T7}:His6-SUMO-FLAG-RodA(A234T)'-GGGSx3-'PBP2*] was generated in a two-piece isothermal assembly reaction with an insert containing RodA(A234T), which was amplified from PR151 with oligonucleotide primers oSS75 and oSS82, and pSS50, which was amplified with oligonucleotide primers oSS74 and oSS83.

pSS60 [*colE1 bla PT7:His6-SUMO-FLAG-RodA(D262A)'-GGGSx3-'PBP2*] was generated in a two-piece isothermal assembly reaction with an insert containing RodA(D262A), which was generated by overlap extension PCR using oligonucleotide primers oSS36 (TTGGTGGATCCATGACGGATAATCCGAATAAAAAAACATTCTGGG), oSS37, oSS96 (ACGCCATACTGCCTTTATCTTCGCGGTACTGGC), and oSS97 (CGAAGATAAAGGCAGTATGGCGTTCGGGGAGAA), and pSS50, which was amplified with oligonucleotide primers oSS74 and oSS83.

pSS62 [*colE1 bla PT7:His6-SUMO-FLAG-RodA(D262A)'-GGGSx3-'PBP2(L61R)*] was generated in a two-piece isothermal assembly reaction with an insert containing RodA(D262A), which was generated by overlapping PCR using oligonucleotide primers

oSS36, oSS37, oSS96, and oSS97, and pSS51, which was amplified with oligonucleotide primers oSS74 and oSS83.

pAAY71 [*aacC1 Psyn135::mCherry*]: To a vector for expressing cytoplasmic mCherry, the mCherry gene was PCR-amplified from pAAY65 [*aacC1 Psyn135::ssdsbA-mCherry*] [53] template using primers oAAY1 (TTTTCATATGTCCAAGGGCGAGGAGGATAACCTG) and oAAY2 (TTTTGTCGACTTATTAGGATCCGCCAGCACCTTTGTAC). The resulting PCR product was digested with NdeI and SalI restriction enzymes and cloned into pAAY65, which was pre-digested with the same enzymes.

References

1. Datsenko KA, Wanner BL. One-step inactivation of chromosomal genes in *Escherichia coli* K-12 using PCR products. Proc Natl Acad Sci USA. 2000;97: 6640–6645. doi:10.1073/pnas.120163297

2. Yu D, Ellis HM, Lee EC, Jenkins NA, Copeland NG, Court DL. An efficient recombination system for chromosome engineering in *Escherichia coli*. Proc Natl Acad Sci USA. 2000;97: 5978–5983. doi:10.1073/pnas.100127597

3. Philippe N, Alcaraz J-P, Coursange E, Geiselmann J, Schneider D. Improvement of pCVD442, a suicide plasmid for gene allele exchange in bacteria. Plasmid. 2004;51: 246–255. doi:10.1016/j.plasmid.2004.02.003

4. Baba T, Ara T, Hasegawa M, Takai Y, Okumura Y, Baba M, et al. Construction of *Escherichia coli* K-12 in-frame, single-gene knockout mutants: the Keio collection. Mol Syst Biol. 2006;2: 2006.0008. doi:10.1038/msb4100050

5. Cho H, Uehara T, Bernhardt TG. Beta-lactam antibiotics induce a lethal malfunctioning of the bacterial cell wall synthesis machinery. Cell. 2014;159: 1300–1311. doi:10.1016/j.cell.2014.11.017

6. Uehara T, Parzych KR, Dinh T, Bernhardt TG. Daughter cell separation is controlled by cytokinetic ring-activated cell wall hydrolysis. EMBO J. 2010;29: 1412–1422. doi:10.1038/emboj.2010.36

7. Gibson DG, Young L, Chuang R-Y, Venter JC, Hutchison CA, Smith HO. Enzymatic assembly of DNA molecules up to several hundred kilobases. Nature Methods. 2009;6: 343–345. doi:10.1038/nmeth.1318

8. Yakhnina AA, McManus HR, Bernhardt TG. The cell wall amidase AmiB is essential for *Pseudomonas aeruginosa* cell division, drug resistance and viability. Mol Microbiol. 2015;97: 957–973. doi:10.1111/mmi.13077

9. Meeske AJ, Riley EP, Robins WP, Uehara T, Mekalanos JJ, Kahne D, et al. SEDS proteins are a widespread family of bacterial cell wall polymerases. Nature. 2016. doi:10.1038/nature19331

10. Guyer MS, Reed RR, Steitz JA, Low KB. Identification of a sex-factor-affinity site in *E. coli* as gamma delta. Cold Spring Harb Symp Quant Biol. 1981;45 Pt 1: 135–140.

11. Bendezú FO, de Boer PAJ. Conditional lethality, division defects, membrane involution, and endocytosis in *mre* and *mrd* shape mutants of Escherichia coli. J Bacteriol. 2008;190: 1792–1811. doi:10.1128/JB.01322-07

12. Cho H, Wivagg CN, Kapoor M, Barry Z, Rohs PDA, Suh H, et al. Bacterial cell wall biogenesis is mediated by SEDS and PBP polymerase families functioning semi-autonomously. Nature Microbiology. 2016;1: 16172. doi:10.1038/nmicrobiol.2016.172

13. Wagner S, Klepsch MM, Schlegel S, Appel A, Draheim R, Tarry M, et al. Tuning *Escherichia coli* for membrane protein overexpression. Proc Natl Acad Sci USA. 2008;105: 14371–14376. doi:10.1073/pnas.0804090105

14. Miller VL, Mekalanos JJ. A novel suicide vector and its use in construction of insertion mutations: osmoregulation of outer membrane proteins and virulence determinants in *Vibrio cholerae* requires *toxR*. J Bacteriol. 1988;170: 2575–2583.

15. Johnson JE, Lackner LL, Hale CA, de Boer PAJ. ZipA is required for targeting of ^{D}MinC/DicB, but not ^{D}MinC/MinD, complexes to septal ring assemblies in *Escherichia coli*. J Bacteriol. 2004;186: 2418–2429. doi:10.1128/JB.186.8.2418-2429.2004

16. Bernhardt TG, de Boer PAJ. Screening for synthetic lethal mutants in *Escherichia coli* and identification of EnvC (YibP) as a periplasmic septal ring factor with murein hydrolase activity. Mol Microbiol. 2004;52: 1255–1269. doi:10.1111/j.1365-2958.2004.04063.x

17. Cherepanov PP, Wackernagel W. Gene disruption in Escherichia coli: TcR and KmR cassettes with the option of Flp-catalyzed excision of the antibiotic-resistance determinant. Gene. 1995;158: 9–14.

18. Yunck R, Cho H, Bernhardt TG. Identification of MltG as a potential terminase for peptidoglycan polymerization in bacteria. Mol Microbiol. Wiley/Blackwell (10.1111); 2016;99: 700–718. doi:10.1111/mmi.13258
